# The SOS response acts as a regulatory switch that balances helicase anti-recombination with transcription–replication conflict resolution activities in *Escherichia coli*

**DOI:** 10.1093/g3journal/jkag205

**Published:** 2026-07-29

**Authors:** Sebastien Fleurier, Ivan Matic

**Affiliations:** Université Paris Cité, CNRS, Inserm, Institut Cochin, F-75014 Paris, France; Université Paris Cité, CNRS, Inserm, Institut Cochin, F-75014 Paris, France

**Keywords:** transcription–replication conflicts, SOS regulon, helicases, double-strand breaks, homologous recombination, quinolones

## Abstract

Transcription–replication conflicts are a major source of genome instability, particularly at highly transcribed ribosomal RNA genes, where RNA polymerase stalling can trigger replication fork collapse and double-strand breaks. We investigated how the SOS DNA damage response regulates the activities of the UvrD, DinG, RecQ, and Rep helicases in *Escherichia coli*, focusing on their roles in resolving transcription–replication conflicts and repairing double-strand breaks at ribosomal RNA loci. We systematically assessed the contribution of each helicase to cell growth, DNA break formation, SOS induction, and survival. Our results show that SOS induction functions as a regulatory switch, modulating UvrD and DinG activities between protective transcription–replication conflict resolution and detrimental anti-recombination. RecA abundance emerges as the key determinant of this switch. While RecQ consistently promotes genome stability by facilitating timely SOS activation and recombinational repair, Rep primarily supports replisome progression, functioning independently of SOS response status. Under nalidixic acid treatment, where survival relies mainly on double-strand break repair, we observed a similar SOS-dependent modulation of helicase activity, reinforcing this regulatory model. Together, these findings uncover a dynamic regulatory mechanism that fine-tunes helicase activities to resolve transcription–replication conflicts while limiting deleterious anti-recombination, thereby preserving chromosome integrity under transcriptional or antibiotic-induced stress.

## Introduction

Efficient and accurate DNA replication is essential for all organisms. Replication fork blockage reduces cellular fitness, destabilizes the genome, and increases mutation and mortality rates. Among the many factors that impede fork progression, increasing evidence shows that transcription-replication conflicts (TRCs) are major natural sources of genomic instability (García-Muse and Aguilera 2016). Conflicts are inevitable because both transcription and replication machineries share the same DNA template and replication moves ∼20 times faster than transcription (Merrikh et al. 2012). RNA polymerase (RNAP) is a major obstacle, especially during head-on encounters. Moreover, even without direct collision, transcription alters DNA topology, generating supercoils and secondary structures such as hairpins and G-quadruplexes (G4) that hinder fork progression. RNAP backtracking or stalling promotes RNA:DNA hybrids (R-loop) formation, exposing single-stranded DNA (ssDNA), leading to fork collapse, DNA breaks, and elevated mutation rates. To counter these threats, bacteria have evolved co-oriented replication and transcription to minimize head-on encounters, particularly for essential genes (Rocha 2004). They also rely on helicases, RNase H enzymes, RNAP modulators, and DNA repair factors to prevent, resolve, and repair the consequences of these harmful conflicts (Merrikh et al. 2012).

Notably, most insights into these processes come from artificial constructs, such as engineered genomic inversions or strongly transcribed genes inserted into chromosomes or plasmids, designed to induce head-on collisions, which are naturally rare and unlikely to occur within essential genes. However, codirectional TRCs do arise in bacterial genomes in highly expressed genes as those coding for ribosomal RNA (*rrn*) (Merrikh et al. 2011). We have previously shown that massive codirectional TRCs spontaneously arise at *rrn* operons in *Escherichia coli* during exit from stationary phase in strains with a reduced number of *rrn* operons and also in wild-type (WT) cells recovering from ribosome-damaging treatments (Fleurier et al. 2022; Fleurier and Matic 2025). In both situations, high *rrn* transcription causes RNAP crowding and stalling, triggering TRCs that lead to replication blockage and DNA breaks at *rrn* loci, SOS induction, increased mutagenesis, and substantial cell death. Our analysis revealed that a *lexA*(Def) allele, which constitutively derepresses the SOS regulon, markedly improves growth and survival, whereas loss of *recA* or *recB* severely compromises both, underscoring the essential role of SOS induction and recombinational repair of double-strand breaks (DSBs). Because the SOS regulon includes about 50 genes, many involved in DNA repair (Simmons et al. 2008), it is likely that functions beyond core DSB repair also help mitigate the deleterious effects of these codirectional TRCs.

In this study, we examined whether 2 DNA helicases, UvrD and DinG, whose expression is controlled by the SOS regulon, impact the outcome of the codirectional TRCs in *E. coli* cells having only one *rrn* operon. UvrD is a superfamily 1 helicase that functions as an ATP-dependent motor moving along ssDNA and unwinding double-stranded DNA (dsDNA) in the 3′→5′ direction (Dillingham 2011). UvrD participates in several DNA repair pathways, including methyl-directed mismatch repair (MMR) (Matson and Robertson 2006), nucleotide excision repair (NER) (Kisker et al. 2013), transcription-coupled repair (TCR) (Pani and Nudler 2017), and recombination. UvrD acts as an anti-recombinase by displacing RecA protein from ssDNA filaments (Veaute et al. 2005; Centore and Sandler 2007; Petrova et al. 2015). UvrD is also recruited to replication forks, where it facilitates replisome progression through the displacement of nucleoprotein complexes (Brüning et al. 2014; Hawkins et al. 2019; Wollman et al. 2024). DinG is a superfamily 2 helicase that likewise functions as an ATP-dependent motor, but it moves along ssDNA and unwinds dsDNA in the 5′→3′ direction (Voloshin et al. 2003). DinG is involved in resolving specific complex DNA structures, such as R-loops and G4, encountered during replication and transcription. DinG can also unwind displacement loops (D-loops) (Voloshin and Camerini-Otero 2007), which are key intermediates formed during homologous recombination and during replication restart following DNA damage. Both UvrD and DinG have been previously shown to play key roles in processing head-on TRCs (Boubakri et al. 2010; Merrikh et al. 2012). The expression of the *dinG* and *uvrD* genes is controlled by the LexA SOS repressor. It was shown that LexA dissociates more rapidly from the *dinG* and *uvrD* operators than from many other LexA-regulated operators, and that these genes belong to a group that is induced early after UV irradiation (Culyba et al. 2018).

Additionally, we investigated the involvement of 2 other helicases, Rep and RecQ, which are not controlled by the SOS regulon but are known to participate in the resolution of head-on TRCs and in rescuing stalled replication forks, respectively (Boubakri et al. 2010). Rep is an ATP-dependent helicase belonging to the SF1 superfamily, which moves along the ssDNA strand and unwinds the dsDNA with 3′→5′ polarity (Dillingham 2011). Rep is involved in replication restart, DNA repair, and recombination. It can remove RNA polymerase complexes that obstruct replication fork progression (Hawkins et al. 2019). At replicative forks, Rep interacts with the replicative DNA helicase DnaB (Guy et al. 2009; Syeda et al. 2019), cooperatively facilitating replication through highly transcribed regions of the chromosome. Rep also prevents RecA from promoting unnecessary recombination during replication fork repair (Mahdi et al. 2006). The RecQ helicase is a superfamily 2 helicase, which is the prototypical member of the RecQ family of helicases (Sharma et al. 2006), and is a crucial guardian of genome stability, primarily operating at the intersection of DNA replication, recombination, and repair. RecQ moves 3′→5′ along the ssDNA track, like UvrD but opposite to DinG. RecQ is an ATP-dependent enzyme that unwinds a broad range of complex DNA structures, including duplex DNA, forked structures, Holliday junctions, D-loops, and G4 DNA (Heyer 2004). RecQ participates in rescuing stalled replication forks by generating ssDNA that provides a substrate for RecA and thus induces SOS and initiates homologous recombination (Hishida et al. 2004). RecQ also suppresses illegitimate recombination in cells with DNA damage, thus preventing deletional chromosomal rearrangements (Hanada et al. 1997).

We found that the SOS induction shifts the activities of UvrD and DinG between potentially harmful anti-recombinase roles and genome-protective TRC-resolving functions at the *rrn* loci. RecQ consistently promotes productive recombination and timely SOS activation, whereas Rep primarily supports basal replisome progression independently of the SOS response. We further examined how these helicases affect the survival of WT cells exposed to the quinolone antibiotic nalidixic acid (NAL), a DNA gyrase inhibitor that generates DSBs and triggers SOS induction, making survival largely dependent on DSB repair (McDaniel et al. 1978; Drlica and Zhao 1997; Aldred et al. 2014). Under these conditions, deletion of *uvrD* or *dinG* improved survival, whereas deletion of *recQ* decreased it, and deletion of *rep* had little effect. Together, these findings reveal that the SOS response not only regulates the expression of the *uvrD* and *dinG* genes but also dynamically balances helicase activities, coordinating replication-facilitating and recombination-inhibiting functions with the cellular need to preserve genome integrity under DNA-damaging stress.

## Materials and methods

### Bacterial strains and growth conditions

All strains used in this study are derivatives of *E. coli* K12 MG1655, which contains the standard 7 *rrn* operons *per* genome and is referred to as the WT in the text (Supplementary Table 1). Mutant derivatives were constructed by P1*vir* transduction of the alleles from either the Keio collection (Baba et al. 2006) or our laboratory collection. Precultures for all experiments were grown at 30 °C for the following reasons: the rRNA synthesis elongation rate of *E. coli* decreases significantly at 30 °C (Zhu et al. 2021); *E. coli* mutants with DNA replication problems, such as *holD*, have improved viability at this temperature (Viguera et al. 2003); and *E. coli* cells growing at lower temperatures have lower base-pair substitutions rates (Foster et al. 2018), which minimizes the likelihood of suppressor mutations arising in strains with a reduced number of *rrn* operons. All experiments were performed using Luria-Bertani (LB) medium (10 g/L NaCl; Difco). When required, the growth medium was supplemented with antibiotics (Sigma-Aldrich) at the following concentrations: Chloramphenicol (Cm) 30 µg ml^−1^, Kanamycin (Kan) 100 µg ml^−1^, and Tetracycline (Tet) 12.5 µg ml^−1^.

### Determination of growth curves

Overnight cultures were diluted 1/1,000 and dispensed into 96-well plates. Each well contained 150 μl of LB medium and was overlaid with 30 μl of mineral oil. During growth at 37 °C with shaking, the optical density at 600 nm (OD_600 nm_) was measured every 2 min over 15 h using a Tecan Infinite M200 PRO microplate reader. The lag time was defined as the time required for OD_600 nm_ after dilution to reach the first intersection point between an extrapolation of the linear portion of the growth curve on a semi-log plot and the growth curve itself. The doubling time was defined as the time required for the bacterial population to increase its OD_600 nm_ from 0.1 to 0.2.

### Quantification of DNA damage

DNA damage was quantified using the Promega Fluorometric DeadEnd Fluorometric TUNEL system. TUNEL is an acronym for terminal deoxynucleotidyl transferase biotin-dUTP nick end labeling. Overnight cultures were diluted 1/1,000 in fresh LB medium and incubated at 37 °C with agitation until they reached an OD_600 nm_ of 0.05. 5 × 10^6^ cells were centrifuged at 5,000 rpm for 10 min at 4 °C, washed twice with cold phosphate-buffered saline (PBS), and resuspended in 0.5 ml of PBS. Cells were fixed with 5 ml of 1% methanol-free formaldehyde on ice for 20 min, then washed twice with cold PBS. Permeabilization was performed using 0.2% Triton X-100 solution for 5 min at room temperature. Cells were washed twice with PBS and stained according to the manufacturer's instructions. Green fluorescence was measured using a Gallios flow cytometer (Beckman Coulter). A minimum of 50,000 cells were analyzed for each time point with a maximum of 2,000 events counted per second.

### Measuring SOS induction

Overnight cultures were diluted 1/1,000 and dispensed into 96-well plates. Each well contained 150 μl of LB medium and was overlaid with 30 μl of mineral oil. Growth and fluorescence measurements were conducted at 37 °C using a Tecan Infinite M200 PRO microplate reader over a 15-h period, with readings taken every 2 min. Growth was monitored by measuring OD_600 nm_. Fluorescence of the SOS response reporter P*_recA_*-*yfp* was monitored with excitation at 490 nm and emission at 530 nm.

P*_recA_*-*yfp* reporter fusion was constructed as follows: The promoter region of *recA* (100 bp upstream of the translational start codon) was amplified from genomic DNA by PCR using the following primers:

*recA* forward (−100 bp upstream of the ATG start codon): 5′GGTCATGACAATTTCTACAAAACACTTG-3′ (BspHI site).

*recA* reverse (−1 bp upstream of the ATG start codon): 5′GCGGTACCTTTTACTCCTGTCATGCCGG-3′ (KpnI site).

The amplified promoter fragment was digested with BspHI and KpnI and cloned upstream of the yellow fluorescent protein (YFP) coding sequence in the pKD3-YFP vector, also digested with the same restriction enzymes. Ligation was performed using standard molecular cloning procedures, generating the transcriptional reporter plasmid P*_recA_*-*yfp*. For the chromosomal integration, the P*_recA_*-*yfp* fusion was integrated into the *intC* locus using λ Red-mediated homologous recombination as described by (Datsenko and Wanner 2000).

The integration cassette was amplified using the following primers containing homology extensions targeting the *intC* locus:

*intC*5′(Forward): 5′CCGTAGATTTACAGTTCGTCATGGTTCGCTTCAGATCGTTGACAGCCGCAGAAGCATTTATCAGGGTTAT-3′.

P*_recA_*-*yfp*3′(Reverse): 5′ATAGTTGTTAAGGTCGCTCACTCCACCTTCTCATCAAGCCAGTCCGCCCAATGGTCCATATGAATATCCT-3′

### Viability assay

Overnight cultures were diluted 1/1,000 in fresh LB medium and incubated at 37 °C with agitation at 150 rpm. At OD_600 nm_ of 0.05, 5 × 10^6^ cells were transferred to clean tubes and centrifuged at 13,000 rpm at 4 °C for 10 min. The pellets were resuspended in PBS containing 1 μM propidium iodide (PI). Samples were incubated for 15 min at room temperature in the dark. Cells were washed in 10^−2^ M MgSO_4_. Analysis was performed using a Gallios flow cytometer (Beckman Coulter). PI fluorescence was measured with a 488 nm excitation laser and 620 nm emission filter. Cells killed by incubation at 90 °C for 5 min were used as a positive control. A minimum of 50,000 cells were analyzed for each time point with a maximum of 2,000 events counted per second.

### Spot test

We first established that the minimal inhibitory concentration (MIC) of NAL for WT cells growing on agar plates is 8 µg ml^−1^. Next, fresh overnight cultures were serially diluted in 10^−2^ mM MgSO_4_. Subsequently, 8 µL from each dilution was spotted onto LB agar plates with or without a sub-inhibitory concentration of NAL, ie 5 µg ml^−1^. After 20 h of incubation at 30 °C, the survival of different strains under these conditions was evaluated by counting colony-forming units (CFUs).

### Determining duration of the growth recovery phase after exposure to nalidixic acid stress

We first established that the MIC of NAL for WT cells growing in liquid LB is 4 µg ml^−1^. Therefore, the MIC for NAL was lower in liquid medium than on agar plates. This difference reflects multiple physical and biological factors associated with the 2 media types. On solid media, cells in colonies experience higher local densities, limited antibiotic diffusion, and steep nutrient and oxygen gradients, which can reduce antibiotic efficacy, such as in biofilms (Hall and Mah 2017). In contrast, well-mixed liquid cultures provide more uniform exposure to antibiotics and better access to nutrients and oxygen. Next, fresh overnight cultures were diluted 1/1,000 in fresh LB medium and incubated in a flask at 37 °C with agitation until they reached an OD_600 nm_ of 0.7. Cells were then diluted 1:100 in fresh LB and incubated for 1 h at 37 °C either without stress or in the presence of the MIC of NAL, ie 4 µg ml^−1^. Survival immediately after treatment was assessed by plating treated and nontreated cells on LB agar and counting CFUs after overnight incubation at 37 °C. To monitor the duration of growth recovery, cells were washed 3 times with LB medium and dispensed into 96-well plates, with each well containing 150 μL of culture overlaid with 30 μL of mineral oil. OD_600 nm_ was measured every 2 min over a 15-h period using a Tecan Infinite M200 PRO microplate reader.

### Statistical analysis

All statistical analyses were performed using GraphPad Prism 8 (GraphPad Software, Inc., La Jolla, CA). Specific details of the statistical tests applied for each experiment are provided in the corresponding figure legends.

## Results

### DNA helicases modulate growth restart, DNA breakage, and viability of cells containing a single *rrn* operon

We first investigated the effects of individually deleting the *dinG*, *uvrD*, *recQ*, and *rep* genes on 2 key growth parameters in *E. coli*: lag phase duration and exponential phase doubling time. These analyses were performed in 2 MG1655 strains: the WT strain carrying all 7 *rrn* operons, and a strain engineered to retain only the *rrnC* operon (hereafter referred to as *rrnC***^+^**), which is prone to RNA polymerase stalling and jamming at the sole remaining *rrn* locus (Fleurier et al. 2022). Stationary phase cultures were diluted into fresh LB medium, and bacterial growth was monitored using a microplate reader.

Deletion of *dinG* or *uvrD* significantly shortened the lag phase of the *rrnC***^+^** strain but had no detectable effect on the exponential phase doubling time, and had no effect on either growth parameter in the WT strain (Fig. 1 and Supplementary Table 2). Deletion of *recQ* significantly prolonged both the lag phase and the exponential phase doubling time of the *rrnC***^+^** strain, while leaving the WT strain unaffected (Fig. 1 and Supplementary Table 2). In contrast, deletion of *rep* did not significantly prolong the lag phase duration, but it increased the exponential phase doubling time of both *rrnC***^+^** and WT strains (Fig. 1 and Supplementary Table 2).

**Fig. 1.**
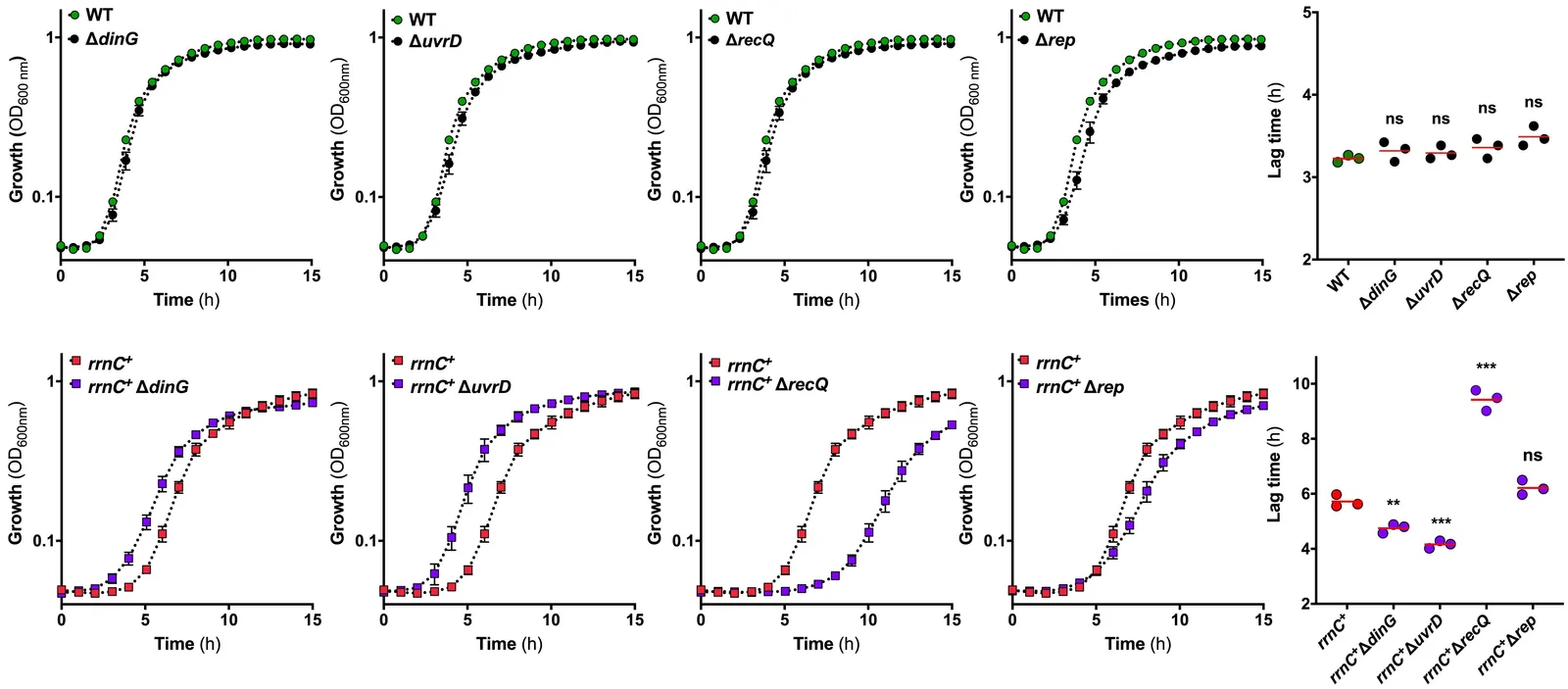
Growth curves of WT and rrnC^+^ strains and their helicase mutant derivatives. Growth curves of the WT strain (upper row), which contains 7 rrn operons; the rrnC^+^ strain with only the rrnC operon (bottom row); and their helicase mutant derivatives. Each data point represents the mean value (±SD) of 3 independent experiments. The panels in the last column show the lag time duration calculated from the growth curves of the WT strain and its mutant derivatives (upper panel) and of the rrnC^+^ strain and its mutant derivatives (bottom panel). Asterisks indicate significant differences relative to the WT (upper panel) or rrnC^+^ (bottom panel) strains according to a *t*-test: **P < 0.01, ***P < 0.001. NS, not significant. Related to Supplementary Table 2.

We previously showed that reducing the copy number of *rrn* operons leads to DNA breakage at remaining *rrn* loci during the lag phase (Fleurier et al. 2022; Fleurier and Matic 2025). Here, we tested whether deletions of the *dinG*, *uvrD*, *recQ*, and *rep* genes affect DNA breakage by using a terminal deoxynucleotidyl transferase dUTP nick end labeling (TUNEL) assay. We quantified DNA breaks in lag phase (OD_600 nm_ of 0.05) WT and *rrnC***^+^** cells. First, we found that deletions of *dinG*, *uvrD*, *recQ*, and *rep* genes had no detectable impact in WT background (Fig. 2a). Second, as we previously reported, the *rrnC***^+^** strain exhibited a 40-fold increase in TUNEL-stained cells compared with the WT (Fig. 2a). Third, we observed that deletion of *dinG* or *uvrD* reduced DNA breakage, deletion of *recQ* increased it, while the *rep* mutant displayed a level of DNA breakage comparable to that of the *rrnC***^+^** strain (Fig. 2a). To confirm that the phenotypes of the Δ*dinG* and Δ*uvrD* mutants were attributable to the loss of the respective helicases, we introduced plasmids carrying functional *dinG* and *uvrD* genes into the *rrnC^+^* Δ*dinG* and *rrnC^+^* Δ*uvrD* strains, respectively. In both cases, complementation restored a level of DNA breakage similar to that observed in the *rrnC***^+^** strain (Supplementary Fig. 1a). We also constructed an *rrnC^+^ uvrD dinG* double helicase mutant and found that this strain exhibited a higher proportion of cells with DNA breakage than the *rrnC^+^* mutant (Supplementary Fig. 1a). These results indicate that UvrD and DinG can partially compensate for each other in resolving TRCs in *rrnC^+^* background. Finally, we observed a strong positive correlation between the duration of the lag phase and the proportion of TUNEL-stained cells across the different *rrnC***^+^** strain derivatives (Fig. 2b). This correlation suggests that the delay in growth resumption is associated with the accumulation of genomic damage.

**Fig. 2.**
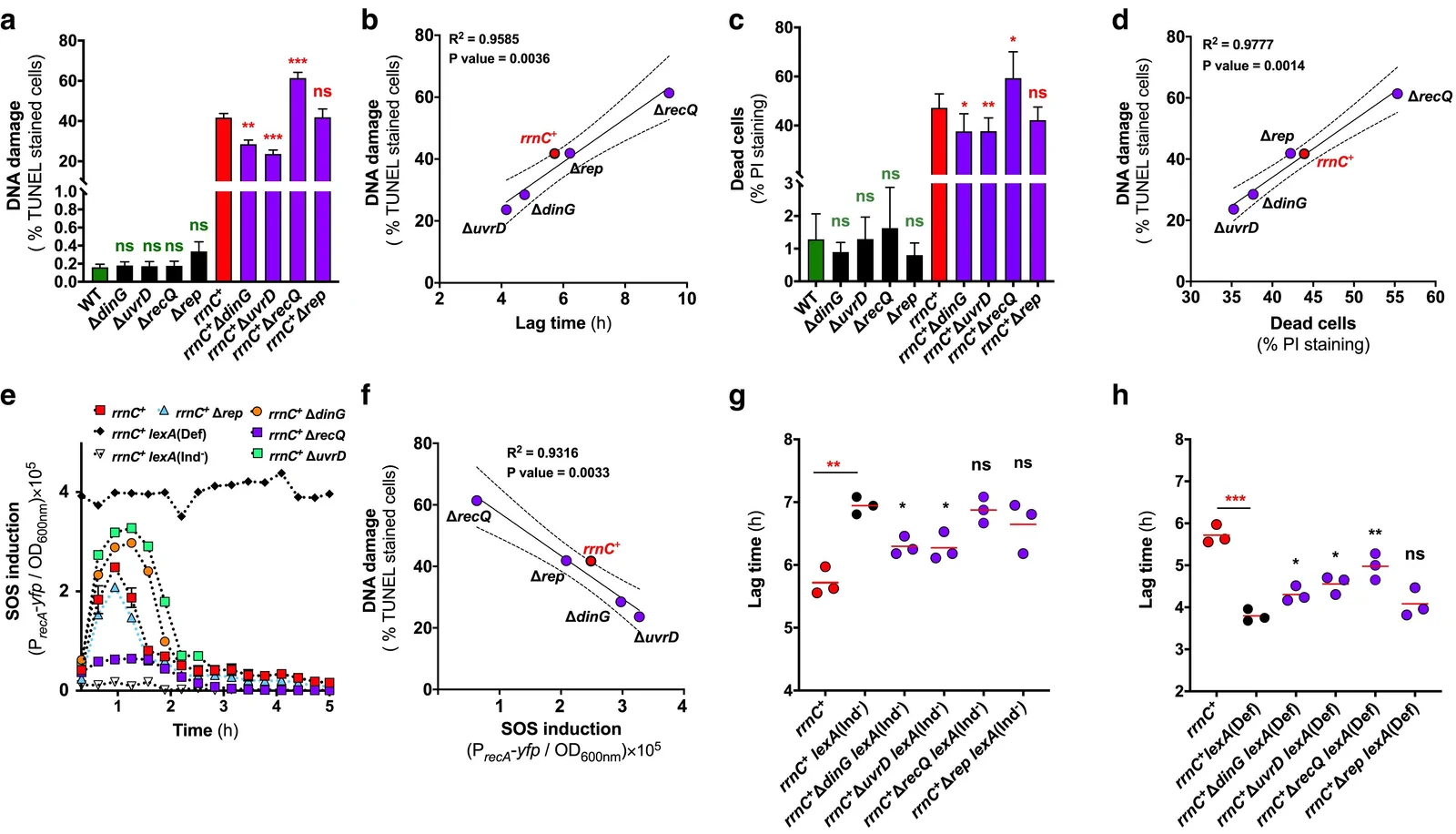
DNA breakage, viability, SOS induction, and growth of rrnC^+^ strain and its helicase mutant derivatives. a) Impact of the deletion of genes encoding helicases on DNA breakage in lag-phase WT and rrnC^+^ strain cells, quantified using a TUNEL assay. Each bar represents the mean value (±SD) of 3 independent experiments. Asterisks indicate significant differences relative to the rrnC^+^ strain according to a *t*-test: **P < 0.01, ***P < 0.001. NS, not significant. b) Linear regression between the extent of DNA breakage and lag phase duration in the rrnC^+^ strain and its helicase mutant derivatives. The dashed line represents the 95% confidence interval. Related to Supplementary Table 2. c) Impact of the deletion of genes encoding helicases on the viability of lag phase WT and rrnC^+^ cells. Dead cells were detected using PI staining and flow cytometry. Each bar represents the mean value (±SD) of 3 independent experiments. Asterisks indicate significant differences relative to the rrnC^+^ strain according to a *t*-test: *P < 0.05, **P < 0.01. NS, not significant. d) Linear regression between the extent of DNA breakage and cell viability in the rrnC^+^ strain and its helicase mutant derivatives. The dashed line represents the 95% confidence interval. e) SOS induction measured using the P*_recA_*-*yfp* reporter in the rrnC^+^ strain, its helicase mutant, *lexA*(Ind^−^) and *lexA*(Def) derivatives. Each data point represents the mean value (±SD) of 3 independent experiments. f) Linear regression between the extent of DNA breakage and the level of SOS induction in the rrnC^+^ strain and its helicase mutant derivatives. The dashed line represents the 95% confidence interval. g) Lag time duration calculated from the growth curves of the different rrnC^+^ strain mutant derivatives (related to Supplementary Table 2). Asterisks indicate significant differences according to a *t*-test: *P < 0.05, **P < 0.01. NS, not significant. Red asterisks indicate comparisons between the rrnC^+^ strain and its lexA(Ind^−^) derivative. Black asterisks indicate comparisons between the *rrnC^+^ lexA*(Ind^−^) strain and its helicase mutant derivatives. h) Lag time duration calculated from the growth curves of the different rrnC^+^ strain mutant derivatives (related to Supplementary Table 2). Asterisks indicate significant differences according to a *t*-test: *P < 0.05, **P < 0.01, ***P < 0.001. NS, not significant. Red asterisks indicate comparisons between the *rrnC^+^* strain and its *lexA*(Def) derivative. Black asterisks indicate comparisons between the *rrnC^+^ lexA*(Def) strain and its helicase mutant derivatives.

We previously showed that reducing the copy number of *rrn* operons leads to massive cell death in the *rrnC***^+^** strain population during the lag phase (Fleurier et al. 2022; Fleurier and Matic 2025). Thus, in this study, we evaluated cell death during the lag phase of cultures of both WT and *rrnC^+^* strains and their helicase mutant derivatives. Cells were stained with PI, which enters dead cells with permeabilized membranes and binds to DNA by intercalating between the bases, but does not enter live cells with intact membranes (Sgorbati et al. 1996). Flow cytometry quantification of dead cells showed that deletions of the *dinG*, *uvrD*, *recQ*, and *rep* genes had no detectable impact on cell viability in the WT background, which had about 1.3% of dead cells (Fig. 2c). However, we observed that deletion of *dinG* or *uvrD* decreased the fraction of dead cells, whereas deletion of *recQ* increased it, while the *rep* mutant displayed a similar fraction to that of the *rrnC***^+^** strain (Fig. 2c). Introduction of functional *dinG* and *uvrD* genes into the *rrnC^+^* Δ*dinG* and *rrnC^+^* Δ*uvrD* strains, respectively, restored the fraction of dead cells to a level comparable to that of the *rrnC***^+^** strain (Supplementary Fig. 1b). The *rrnC^+^ uvrD dinG* double helicase mutant exhibited a higher proportion of dead cells than the *rrnC^+^* mutant (Supplementary Fig. 1b). Finally, we observed a strong positive correlation between the number of dead cells and the proportion of TUNEL-stained cells across the different *rrnC***^+^** strain derivatives (Fig. 2d). This correlation suggests that cell death is associated with the accumulation of genomic damage.

Together, these results demonstrate that DNA helicases differentially modulate growth restart, genome stability, and viability of *rrnC*^+^ cells. In particular, DinG and UvrD increase lag phase-associated DNA breakage and delay growth resumption, RecQ promotes genome stability and facilitates growth resumption, whereas Rep has no significant impact on either phenotype.

### Impact of SOS induction on the DNA helicases-dependent modulation of the growth restart of the ***rrnC*^+^** strain

Next, we sought to examine in more detail the detrimental effects of the UvrD and DinG helicases on *rrnC*^+^ cells during growth restart, a phase characterized by strong induction of the SOS response (Fleurier et al. 2022). SOS induction enhances the cellular capacity for homologous recombination and increases the expression of the *uvrD* and *dinG* genes. We previously showed that RecA- and RecB-dependent homologous recombination repairs dsDNA breaks that arise from codirectional TRCs (Fleurier et al. 2022). However, both UvrD and DinG have the potential to interfere with homologous recombination by removing RecA protein (Veaute et al. 2005) and unwinding recombination DNA intermediates (Voloshin and Camerini-Otero 2007). The interplay between the examined helicases and SOS regulon is further complicated by the fact that deletion of *uvrD* or *rep* triggers a weak spontaneous SOS response in WT *E. coli* cells (Ossanna and Mount 1989; O’Reilly and Kreuzer 2004), whereas RecQ helicase can promote SOS induction by unwinding DNA at the replication fork and generating ssDNA (Hishida et al. 2004).

We tested whether deletions of genes coding for 4 helicases affect SOS induction in *rrnC*^+^ cells using a P*_recA_*-*yfp* reporter (Fig. 2e). As a control, we used *rrnC*^+^ strain carrying *lexA*(Def) and *lexA*(Ind^−^) alleles. The *lexA*(Def) corresponds to a *lexA* gene deletion that constitutively derepresses all LexA-controlled genes. The *lexA*(Ind^−^) allele (Mount et al. 1972) encodes a noncleavable LexA protein that prevents the induction of all LexA-controlled genes while maintaining their basal expression (Courcelle et al. 2001). The P*_recA_*-YFP reporter was induced in *rrnC*^+^ cells only during the growth restart phase, as previously reported (Fleurier et al. 2022). *lexA*(Def) caused strong constitutive expression, and *lexA*(Ind^−^) abolished it throughout all growth phases. Δ*uvrD* or Δ*dinG* alleles increased reporter induction, while Δ*rep* slightly decreased it relative to the *rrnC*^+^ cells. Reporter expression was strongly suppressed by the Δ*recQ* allele. Crucially, there was a strong negative correlation between the level of the P*_recA_*-*yfp* reporter induction and the proportion of TUNEL-stained cells (Fig. 2f). This implies that the ability of a strain to induce SOS is the primary determinant of DSB repair and survival.

To avoid potential confounding effects that may arise from the impact from deletions of genes encoding the helicases on SOS induction, we combined deletions of these genes with either a *lexA*(Ind^−^) or *lexA*(Def) allele and measured lag phase duration (Fig. 2, g and h, Supplementary Fig. 2, and Supplementary Table 2). As previously reported (Fleurier et al. 2022), introducing the *lexA*(Ind^−^) or *lexA*(Def) allele significantly increased and decreased, respectively, the lag phase of the *rrnC***^+^** strain (Fig. 2, g and h). In the *lexA*(Ind^−^) background, deletion of *dinG* or *uvrD* significantly decreased the lag phase, whereas deletion of *rep* or *recQ* had no effect (Fig. 2, g and h, Supplementary Fig. 2, and Supplementary Table 2). In contrast, in the *lexA* (Def) background, deletion of *dinG*, *uvrD*, or *recQ* significantly increased the lag phase, suggesting that while heightened recombination capacity under high SOS induction can resolve DSBs, unresolved TRCs accumulate in the absence of these helicases (Fig. 2, g and h, Supplementary Fig. 2, and Supplementary Table 2). Finally, deletion of *rep* had no effect in the *lexA*(Def) background (Fig. 2, g and h, Supplementary Fig. 2, and Supplementary Table 2).

These results indicate that the effects of UvrD and DinG on growth restart in *rrnC*^+^ cells are strongly modulated by the level of SOS induction, exhibiting divergent trends in *lexA*(Ind^−^) and *lexA*(Def) backgrounds. We propose that this primarily reflects the interplay between UvrD and DinG helicases’ activities, and homologous recombination capacity across the *lexA* genotypes analyzed in this study. RecQ promotes SOS induction and contributes to efficient growth restart in an SOS-dependent manner. Rep does not play a role in any *lexA* background.

### Impact of SOS induction and DNA helicases on survival of nalidixic acid-treated cells

DNA helicases are essential for resolving TRCs by removing obstacles that impede replication fork progression. However, certain helicases, such as UvrD and DinG, might potentially interfere with homologous recombination-mediated repair of TRC-induced DSBs. To disentangle these opposing activities, we exposed the WT strain and its various mutant derivatives to NAL, making survival largely dependent on DSB repair capacity. We performed 2 types of experiments: growth of cells on LB plates containing 5 µg/ml NAL (Fig. 3), and growth recovery in liquid LB medium after treatment with 4 µg/ml NAL (Fig. 4). We used concentrations of NAL that affect DNA synthesis without significantly impacting RNA synthesis (Crumplin and Smith 1975).

**Fig. 3.**
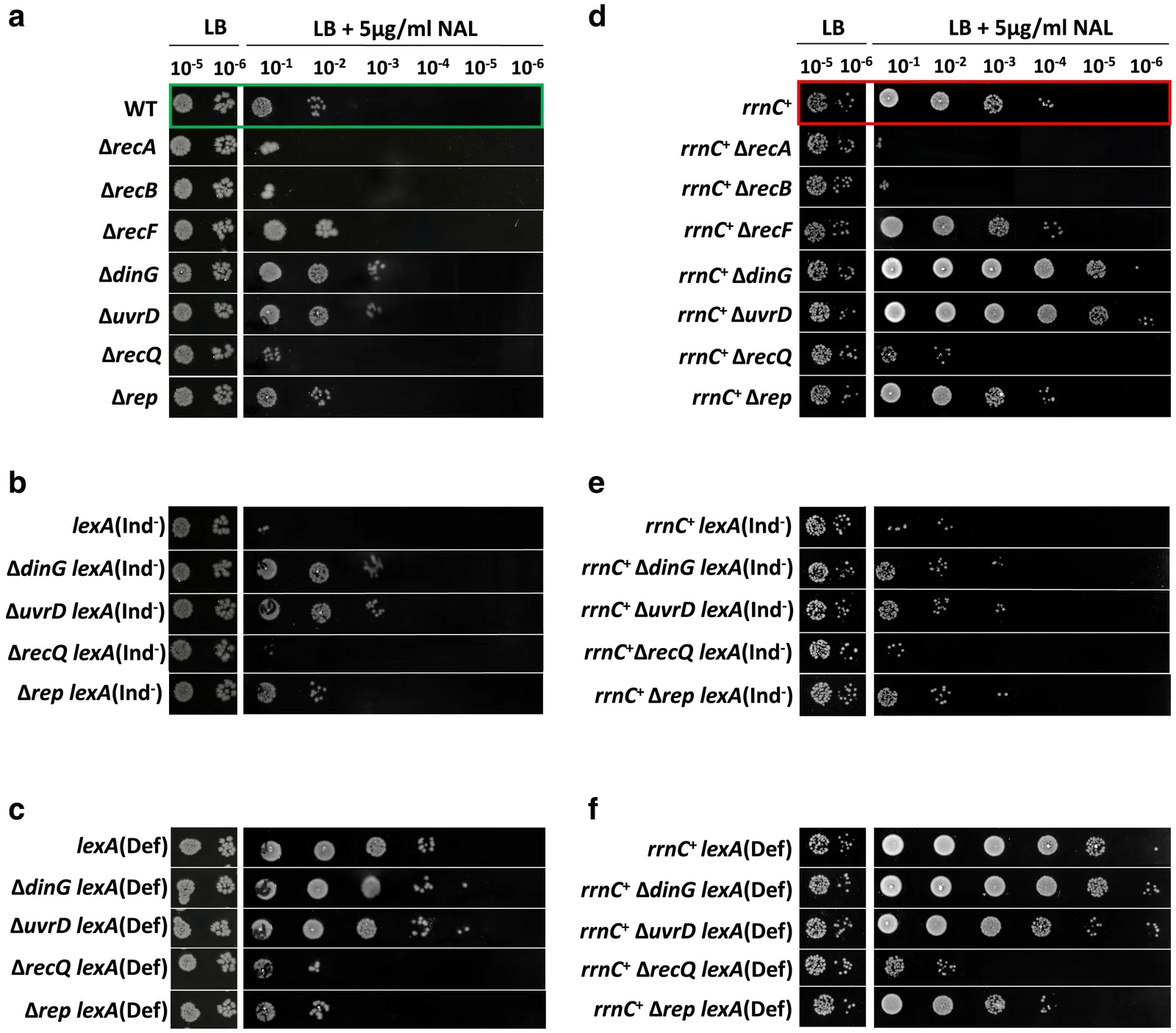
Survival of WT and *rrnC^+^* strains and their mutant derivatives treated with nalidixic acid. Overnight liquid cultures were serially diluted and spotted onto LB agar plates containing or not containing 5 µg/ml NAL. Mutant derivatives shown are: a) WT, b) lexA(Ind^−^), c) lexA(Def), d) *rrnC*^+^, e) *rrnC*^+^, e) *rrnC*^+^ lexA(Ind^−^), f) *rrnC*^+^ lexA(Def). The results are representative of at least 3 independent experiments.

**Fig. 4.**
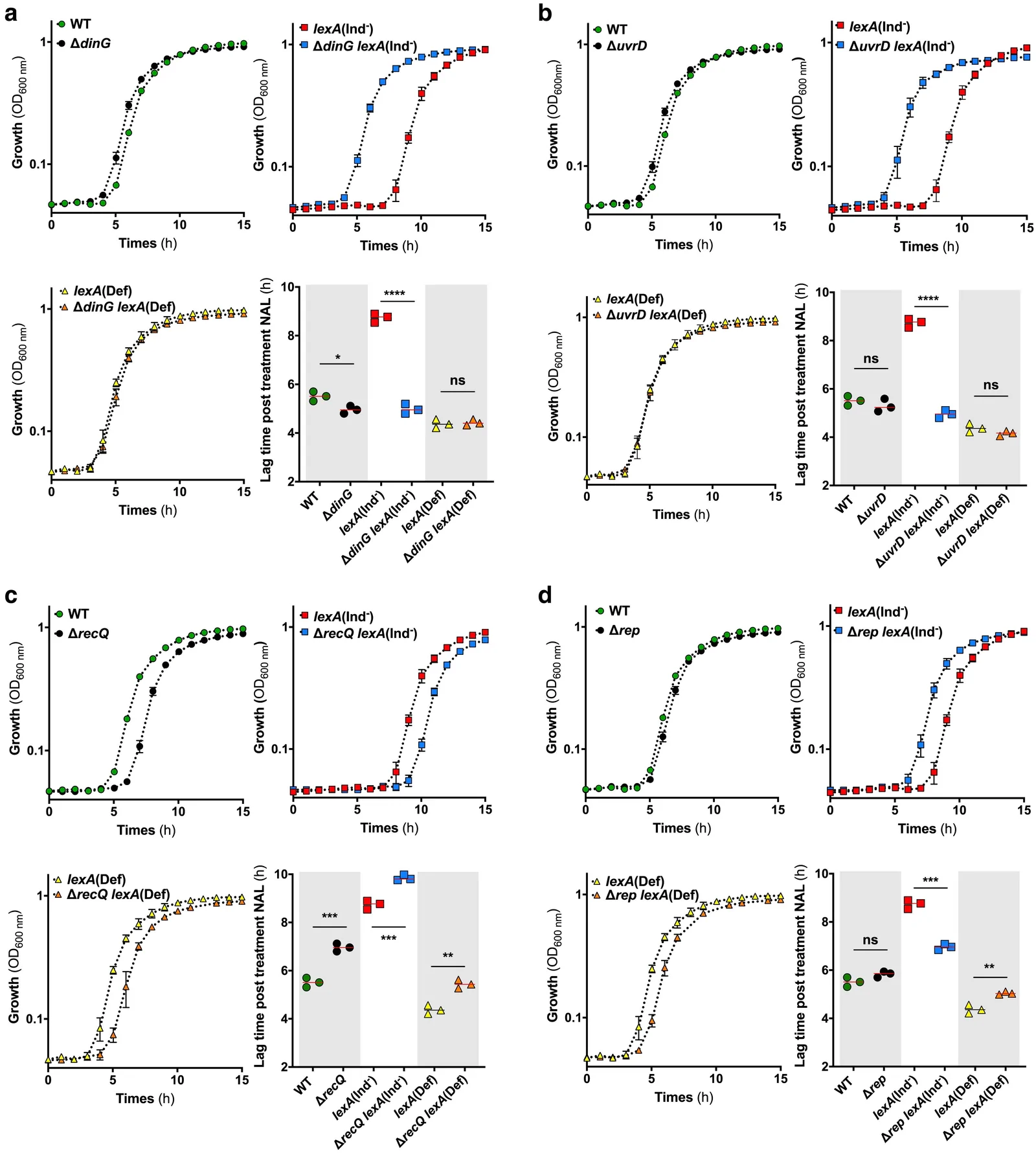
Growth resumption of WT and its mutant derivatives after nalidixic acid treatment. Exponentially growing cells were exposed to 4 µg/ml NAL for 1 h, washed, and regrowth was monitored in fresh liquid LB medium without NAL. Deletions of the helicase-encoding genes a) *dinG*, b) *uvrD*, c) *recQ*, and d) *rep* were introduced into WT, *lexA*(Ind^−^), and *lexA*(Def) strains. The growth restart duration was calculated from the growth curves of all these strains. Each data point represents the mean value (±SD) of 3 independent experiments. Asterisks indicate significant differences according to a *t*-test: **P < 0.01, ***P < 0.001, ****P < 0.0001. NS, not significant.

First, serial dilutions of cells were spotted onto LB plates containing a sub-inhibitory concentration of NAL, and growth/survival was assessed after overnight incubation. Exposure to 5 µg/ml NAL reduced the survival of WT cells by approximately 10^4^-fold relative to the untreated control (Fig. 3a). Deletion of *recA* or *recB* further reduced survival, whereas deletion of *recF* had no effect relative to WT (Fig. 3a). Introducing *lexA* (Ind^−^) or *lexA*(Def) alleles decreased or increased survival relative to WT, respectively (Fig. 3, a to c). These results confirm that RecA- and RecB-mediated DSB repair and SOS induction are necessary for the survival of NAL-treated cells. Deletions of *dinG* and *uvrD* improved survival relative to WT, *recQ* deletion decreased survival, and *rep* deletion had no effect (Fig. 3a). By combining *lexA*(Ind^−^) allele with deletions of genes coding for DNA helicases, we observed that *recQ* deletion had no effect, whereas *rep, uvrD,* and *dinG* deletions increased survival to varying extents (Fig. 3b). Strains carrying *lexA*(Ind^−^) *uvrD* or *lexA*(Ind^−^) *dinG* alleles survived even better than WT, whereas survival of the *lexA*(Ind^−^) *rep* strain was comparable to that of WT (Fig. 3, a and b). By combining *lexA*(Def) allele with deletions of genes coding for DNA helicases, we observed that: *uvrD* and *dinG* deletions had no effect, whereas *recQ* and *rep* deletions reduced survival (Fig. 3c).

Second, we repeated this experiment using different *rrnC*^+^ strain derivatives. This experiment was motivated by our observation that *E. coli* strains carrying decreasing numbers of *rrn* operons (7 in WT, 6, 4, 2, or 1 in *rrnC^+^*) exhibited progressively higher survival on plates containing 5 µg/ml NAL (Supplementary Fig. 3a), in line with previous results obtained with another quinolone antibiotic ciprofloxacin (Bollenbach et al. 2009). For example, we found that survival of the *rrnC*^+^ strain was 10^2^-fold higher than that of WT. Because we previously showed that decreasing the number of *rrn* operons results in increasing levels of SOS induction (Fleurier et al. 2022), we hypothesized that this enhanced survival reflects improved DSB repair capacity driven by elevated SOS activity. To test this, we introduced the *lexA*(Ind^−^) allele into these strains and found that the correlation between *rrn* operon number and NAL survival was completely abolished (Supplementary Fig. 3a), confirming our hypothesis.

Introduction of Δ*recA*, Δ*recB*, or *lexA*(Ind^−^) alleles strongly diminished survival of the *rrnC*^+^ strain, whereas *lexA*(Def) increased survival, and Δ*recF* had no effect (Fig. 3, d to f). When *rrnC*^+^ was combined with deletions of genes coding for DNA helicases, we observed that Δ*recQ* decreased survival, Δ*rep* had no effect, and Δ*uvrD* or Δ*dinG* increased survival (Fig. 3d). Introduction of functional *dinG* and *uvrD* genes into the *rrnC^+^* Δ*dinG* and *rrnC^+^* Δ*uvrD* strains, respectively, restored the NAL-induced cytotoxicity to a level comparable to that of the *rrnC***^+^** strain (Supplementary Fig. 3b). When *rrnC*^+^  *lexA*(Ind^−^) was combined with deletions of genes coding for DNA helicases, Δ*recQ* did not further diminish survival, whereas Δ*rep,* Δ*uvrD,* or Δ*dinG* increased survival (Fig. 3e). Combining the *rrnC*^+^  *lexA*(Def) strain with deletions of genes encoding Δ*uvrD*, Δ*rep*, and Δ*recQ* DNA helicases decreased survival in an increasing order, whereas Δ*dinG* caused a mild increase in survival (Fig. 3f).

Finally, exponentially growing cells were exposed to the MIC of NAL, ie 4 µg/ml, in liquid LB for 1 h, washed, and their survival was assessed by plating treated and untreated cells on LB agar and counting CFUs after overnight incubation at 37 °C. Among all tested strains, only the *lexA*(Def) Δ*recQ* mutant survived significantly less compared to WT (Supplementary Table 3). In parallel, the resumption of growth of the different strains after NAL treatment was monitored in fresh liquid LB medium. Deletion of the *dinG* gene did not significantly affect the exponential growth of WT, *lexA*(Ind^−^), or *lexA*(Def) strains after NAL treatment (Fig. 4a and Supplementary Table 4). However, it slightly reduced the posttreatment growth restart delay relative to WT, greatly reduced that of *lexA*(Ind^−^), and had no impact on that of *lexA*(Def) (Fig. 4a and Supplementary Table 4). Deletion of the *uvrD* gene produced phenotypes identical to those observed upon deletion of the *dinG* gene across all tested conditions and genotypes (Fig. 4b and Supplementary Table 4). Deletion of the *recQ* gene did not significantly affect the exponential growth of WT, *lexA*(Ind^−^), or *lexA*(Def) strains after NAL treatment (Fig. 4c and Supplementary Table 4). However, it increased the posttreatment growth restart delay in WT, *lexA*(Ind^−^), and *lexA*(Def) strains (Fig. 4c and Supplementary Table 4). Finally, deletion of the *rep* gene did not significantly increase the posttreatment growth restart delay in WT, but it caused pronounced increase in *lexA*(Def), and reduced the delay in *lexA*(Ind^−^) (Fig. 4d and Supplementary Table 4).

In conclusion, *uvrD* and *dinG* deletions enhance growth restart and survival after NAL treatment in an SOS-dependent manner, *rep* deletion has genetic background-specific effects, and *recQ* deletion consistently impairs recovery. Overall, SOS induction level dictates whether these helicases are protective or detrimental under NAL-induced DNA damage.

### Impact of RecA levels on helicase anti-recombination activity and recombination-mediated DNA repair

The results above show that the effects of DNA helicases on cell survival and growth recovery strongly depend on the level of SOS induction. In particular, the detrimental effects of UvrD and DinG observed on growth restart in *rrnC*^+^ cells or after NAL treatment were alleviated when SOS induction was constitutively increased by deleting the gene encoding the LexA repressor [*lexA*(Def)] and exacerbated when it was prevented by a mutation that renders LexA noncleavable [*lexA*(Ind^−^)]. Because SOS activation increases RecA levels and promotes homologous recombination-mediated DSB repair (Bell and Kowalczykowski 2016), these observations suggest that the balance between helicase anti-recombination activity and homologous recombination-mediated repair may largely depend on RecA abundance. To test this directly, we used a constitutive operator *recAo^c^* allele that is no longer repressed by LexA (Ginsburg et al. 1982). In the *lexA*(Ind^−^) *recAo^c^* background, all LexA-controlled genes are repressed, but RecA accumulates at high levels, enabling us to assess whether elevated RecA alone can counteract helicase anti-recombination activity and promote homologous recombination-mediated DNA repair.

Serial dilutions of cells were spotted onto LB plates containing 5 µg/ml NAL, and growth/survival was assessed after overnight incubation. Introduction of the *recAo^c^* allele did not change survival of WT, Δ*dinG*, and Δ*uvrD* strains (Figs. 3a and 5), which is expected because NAL treatment induces SOS and, therefore, the *recA* gene expression in all these strains. However, the *recAo^c^* allele improved survival of the Δ*recQ* strain (Figs. 3a and 5), indicating that overproduction of RecA protein is sufficient to compensate for the loss of SOS induction resulting from deletion of the *recQ* gene (Fig. 2e). Introduction of the *recAo^c^* allele restored survival of the *lexA*(Ind^−^) and *lexA*(Ind^−^) Δ*recQ* strains to WT levels, did not change that of *lexA*(Ind^−^) Δ*rep* strain, and increased survival of the *lexA*(Ind^−^) Δ*dinG* and *lexA*(Ind^−^) Δ*uvrD* strains above WT levels (Figs. 3b and 5).

**Fig. 5.**
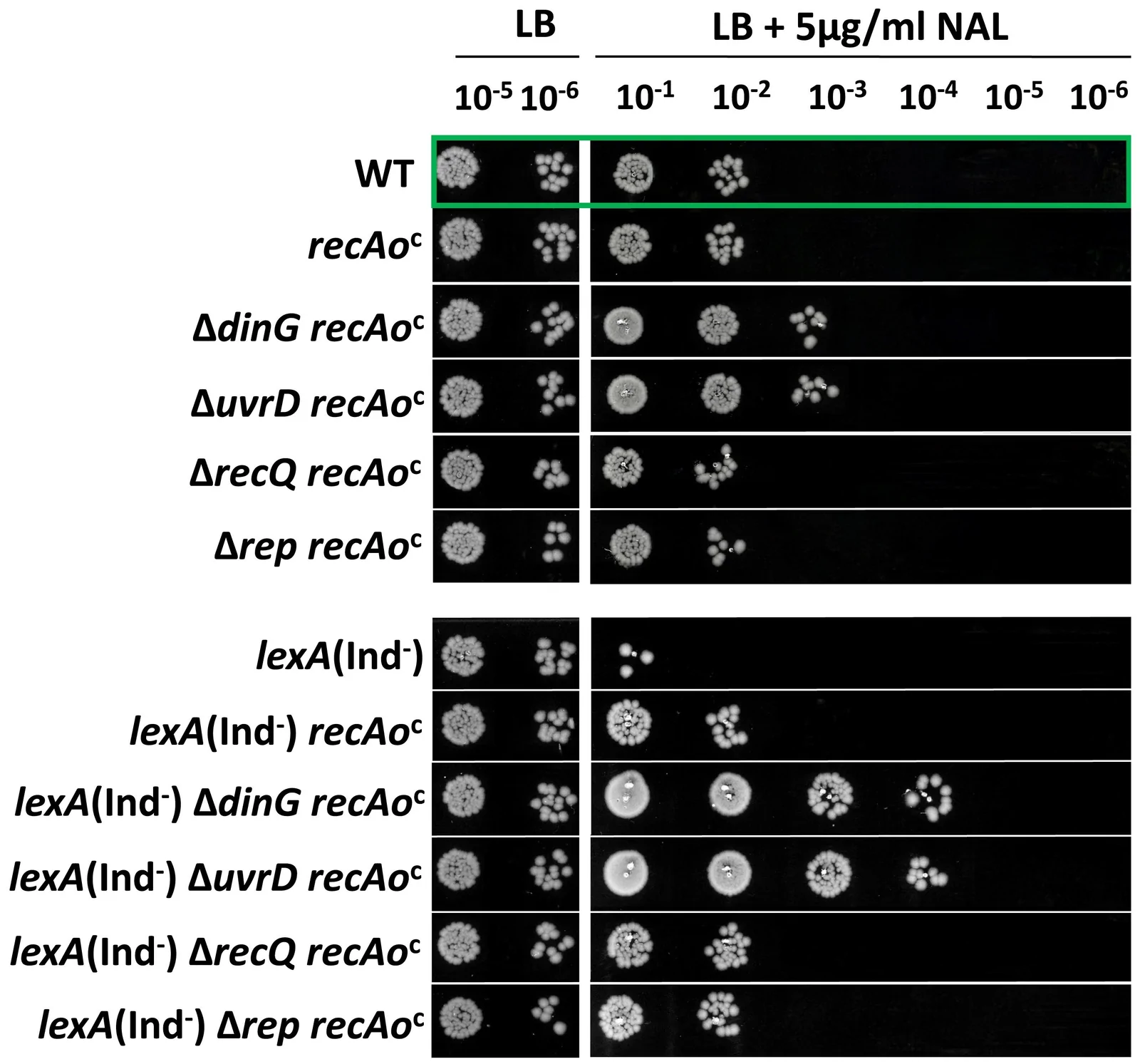
Impact or *recAo^c^* allele on survival of WT and *lexA*(Ind^−^) strains and their mutant derivatives treated with nalidixic acid. Overnight liquid cultures were serially diluted and spotted onto LB agar plates containing or not containing 5 µg/ml NAL. The results are representative of at least 3 independent experiments.

Together, these results indicate that RecA abundance is a key determinant of the balance between helicase-mediated anti-recombination and recombination-mediated DNA repair. Increasing RecA levels is sufficient to overcome helicase anti-recombination activity and restore efficient DNA repair when SOS induction is blocked.

## Discussion

Transcription–replication conflicts are major endogenous sources of genome instability across bacteria and eukaryotes (Merrikh et al. 2012; García-Muse and Aguilera 2016). Their severity depends on transcriptional intensity, collision orientation, RNAP backtracking, R-loop stabilization, and the efficiency of accessory proteins and pathways that process TRCs. We have previously shown that in *E. coli*, rrn operons, which encode ribosomal RNA, constitute major TRC hotspots due to their very high transcriptional output and dense RNAP clustering (Fleurier et al. 2022; Fleurier and Matic 2025). Resolution of codirectional TRCs at *rrn* operons requires dismantling of R-loops by RNase H, MazF-mediated ribosomal RNA cleavage, and removal of stalled or backtracked RNAPs by the transcription factors Mfd, RapA, and GreA (Fleurier et al. 2022; Fleurier and Matic 2025). When unresolved, TRCs generate DSBs, genome degradation, and ultimately cell death (Fleurier et al. 2022; Fleurier and Matic 2025). Cell survival therefore relies heavily on the capacity to repair DSBs through the SOS response induction and the RecA-RecB recombination pathway. Here we examined the roles of 4 DNA helicases, DinG, UvrD, RecQ, and Rep, in TRC resolution and DSB repair. Our results show that the outcome of TRCs at *rrn* operons is determined by how SOS induction modulates helicase abundance and activity, producing idiosyncratic helicase contributions across different levels of the SOS response (Fig. 6, Supplementary Table 5). This regulatory balance is mirrored during treatment with sub-inhibitory concentration of NAL, which impedes DNA replication and generates DSBs.

**Fig. 6.**
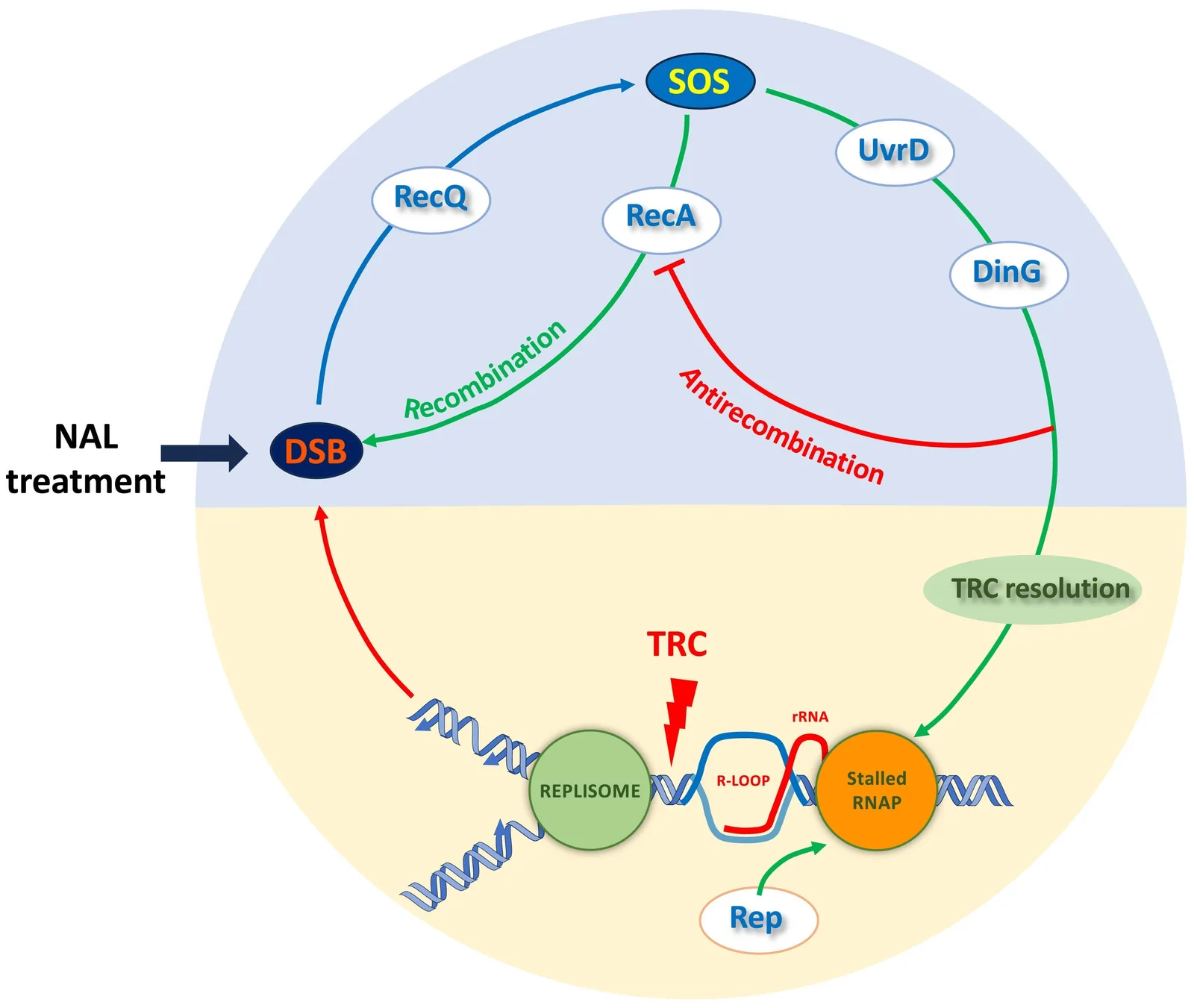
Schematic model illustrating how codirectional transcription–replication conflicts (TRCs), formed at highly expressed *rrn* loci during lag phase exit, are resolved. RNA polymerase stalling at *rrn* loci leads to R-loop formation, replisome arrest, and double-strand breaks (DSBs). These DSBs trigger SOS induction, a process promoted by the RecQ helicase through the generation of ssDNA for RecA nucleation, thereby increasing RecA levels to support recombinational DSB repair. However, SOS induction also upregulates the UvrD and DinG helicases. While UvrD and DinG contribute to TRC resolution and thus limit further DSB formation, they can also act as anti-recombinases by dismantling RecA filaments and unwinding D-loops, thereby inhibiting recombinational repair. At low levels of SOS induction, the anti-recombination activities of UvrD and DinG predominate. Conversely, high SOS induction elevates RecA abundance sufficiently to overcome this inhibition and promote recombination. Rep supports replisome progression independently of SOS, and does not contribute to the resolution of codirectional TRCs. Treatment with nalidixic acid (NAL) recapitulates this balance between the level of SOS induction, DSB repair and helicase-mediated anti-recombination activities (shown in the blue upper semi-circle). NAL stabilizes DNA gyrase–DNA cleavage complexes, which are converted into DSBs upon collision with advancing replisomes. Survival following NAL treatment therefore requires robust SOS activation, the attenuation of helicase anti-recombination activities, and efficient recombination-mediated repair. All information relevant to understanding our model can be found in Supplementary Table 5, which compiles the functional effects of UvrD, DinG, RecQ, and Rep in different genetic backgrounds across growth restart, DNA breakage, cell viability, SOS induction, and nalidixic acid survival.

### The dual role of UvrD and DinG in TRC resolution and recombination

UvrD and DinG helicases are traditionally regarded as enzymes that promote replication fork progression by resolving disruptive nucleic acid structures and displacing obstructive protein complexes, thereby maintaining coordination between replication and transcription (Boubakri et al. 2010; Merrikh et al. 2012). Our findings reveal that they can also be detrimental during TRCs at highly transcribed *rrn* loci, where recombinational repair of DSBs is essential for genome integrity and survival. Deletion of either the *uvrD* or *dinG* genes shortened the lag phase, reduced DNA breakage, increased viability of *rrnC*^+^ cells, and improved survival during NAL treatment, which strongly depends on DSB repair (Fig. 6). These benefits disappeared in *lexA*(Def) cells, where the SOS regulon is constitutively derepressed and recombination capacity is maximal, indicating that the harmful effects of UvrD and DinG may mainly arise from anti-recombination activity. This aligns with previous studies showing that UvrD dismantles RecA nucleofilaments and limits homologous recombination (Veaute et al. 2005; Centore and Sandler 2007; Petrova et al. 2015). DinG unwinds RecA-formed D-loops (Voloshin et al. 2003; Voloshin and Camerini-Otero 2007) and can therefore destabilize recombination intermediates required for fork restart and DSB repair. Under limited SOS induction, low RecA abundance likely renders recombination intermediates more susceptible to helicase-mediated anti-recombination, thereby impairing DSB repair.

### RecQ as a guardian of replication recovery and genome integrity

RecQ helicase displayed a universally protective role in all assays, consistently reducing DNA breakage and cell death, and promoting growth recovery both after stationary phase and during subinhibitory NAL treatment (Fig. 6). These phenotypes align with RecQ known functions in generating ssDNA substrates for RecA loading and facilitating SOS induction (Hishida et al. 2004), as well as in suppressing aberrant recombination events (Hanada et al. 1997). RecQ, together with the RecJ exonuclease, is also implicated in processing stalled or reversed replication forks, thereby channeling repair intermediates into productive homologous recombination pathways (Heyer 2004; Michel et al. 2007; Morimatsu and Kowalczykowski 2014). This function may be particularly important at *rrn* loci, where fork regression and the processing of complex DNA structures are likely recurrent.

Unlike deletion of the *uvrD* or *dinG* genes, deletion of *recQ* increased the lag phase and DNA breakage, decreased SOS induction and the viability of *rrnC*^+^ cells, and sensitized cells to the DSB-inducing agent NAL regardless of the *lexA* background. We therefore view RecQ as a helicase that promotes productive recombination and prevents pathological fork-processing outcomes. The negative correlation between DNA breakage and SOS induction across *rrnC*^+^ derivatives supports the model that RecQ promotes timely SOS activation and recombinational repair, primarily through the induction of high RecA levels. In its absence, both processes are compromised, thus increasing the incidence of lethal DSBs. Consistent with this model, elevating RecA levels through the *recAo^c^* allele restored the survival of Δ*recQ* strains during NAL treatment even in the *lexA* (Ind^−^) strain, indicating that RecQ contributes to survival largely by enabling efficient RecA accumulation. For this reason, we favor the possibility that recombination-mediated repair is a major contributor to the positive role of the SOS response induction, rather than other SOS-regulated factor(s).

### Rep helicase: general fitness factor rather than TRC-resolution specialist

Our findings indicate that Rep primarily supports replication progression (Fig. 6), as deletion of *rep* does not affect lag phase duration, DNA breakage, or cell death, but increases the exponential phase doubling time in both WT and *rrnC*^+^ strains. This is consistent with Rep's established role in cooperating with the replicative helicase DnaB to promote fork progression through protein-bound DNA obstacles (Guy et al. 2009; Atkinson et al. 2011). This is further supported by the observation that deletion of the *rep* gene increases the time required to replicate the chromosome (Atkinson et al. 2011). Unlike UvrD or DinG, whose phenotypes shift dramatically across LexA backgrounds, the impact of Rep remains largely unaffected by SOS status. This suggests that Rep does not play a significant role in the SOS-stimulated resolution of TRCs or in the repair of TRC-induced DSBs at *rrn* loci.

However, the impact of *rep* deletion on NAL susceptibility shows a clear dependence on the *lexA* background: no effect in *lexA*^WT^, increased survival in *lexA*(Ind^−^), and decreased survival in *lexA*(Def). Quinolones such as NAL act by stabilizing DNA gyrase–DNA cleavage complexes, which stall replication forks and are converted into double-strand breaks when collided with by replisomes (Aldred et al. 2014). Rep has been proposed to either displace quinolone-stabilized gyrase cleavage complexes or assist in replication restart after resolution of quinolone-induced replication fork blockage (Henderson and Kreuzer 2015). Our data support the first scenario: Rep removes the quinolone-stabilized gyrase complex bound to cleaved DNA, allowing repair and replication to proceed, but this also exposes cleaved DNA that can be converted into DSBs if replisomes encounter it before ligation. This effect is buffered in *lexA*(Def) cells but persists in *lexA*(Ind^−^) cells, where DSB repair capacity is limited. By contrast, the second scenario—where Rep primarily functions in replication restart—would be expected to negatively affect all *lexA* backgrounds, which does not align with our observations.

### Integration of SOS induction, TRC resolution, and repair pathway choice

The SOS-dependent helicase functional shift reflects a fundamental evolutionary trade-off in the maintenance of the bacterial genome. Anti-recombinases such as UvrD limit illegitimate or homeologous recombination that could otherwise generate deleterious genomic rearrangements during normal growth. Consistent with this view, deletion of the *uvrD* gene confers a spontaneous hyper-recombination phenotype (Zieg et al. 1978; Arthur and Lloyd 1980; Bierne et al. 1997). However, when DSBs are generated at high levels, such as by *rrn*-associated TRCs, recombination becomes essential for cell survival. In this context, anti-recombination activity becomes maladaptive, compromising survival until SOS-driven upregulation of recombination factors restores DSB repair proficiency. Thus, the LexA-regulated recombination landscape dynamically calibrates helicase activity to match the prevailing DNA damage burden.

UvrD exemplifies this trade-off mechanistically. Deletion of the *uvrD* gene renders cells sensitive to DNA damaging agents and hyper-mutagenic, because UvrD is essential for NER and MMR (Matson and Robertson 2006; Kisker et al. 2013), respectively. It is therefore plausible that the anti-recombinase activity of UvrD is essential for preventing unscheduled or toxic recombination events triggered by the ssDNA gaps generated by UvrD during both NER and MMR. However, this activity becomes harmful when high recombination capacity is required for cell survival during intense TRCs or NAL treatment. This mechanistic trade-off is conserved for UvrD homologs in other bacterial species, as well as in eucaryotes. For example, *Helicobacter pylori* UvrD helicase contributes to the repair of damaged DNA and suppresses homologous intragenic recombination (Kang and Blaser 2006). *Bacillus subtilis* PcrA helicase, which shares 42% sequence identity with *E. coli* UvrD, is involved in UV repair, counteracts the toxic effects of the RecFOR complex at replication forks, and inhibits RecA-dependent strand exchange *in vitro* (Lestini and Michel 2007; Dillingham 2011). Inactivation of the SRS2 gene, which encodes the UvrD-homologous Srs2 helicase in *Saccharomyces cerevisiae*, increases sensitivity to genotoxic agents and leads to toxic hyper-recombination (Broomfield 2002; Fabre et al. 2002; Schmidt and Kolodner 2006; Elango et al. 2017). Consistent with UvrD function, Srs2 inhibits recombination by disrupting Rad51, the eukaryotic homolog of RecA, nucleoprotein filaments (Krejci et al. 2003; Veaute et al. 2003).

Bacterial genome stability networks, therefore, operate under a dynamic equilibrium in which helicases with overlapping substrate specificities occupy context-dependent roles. RecQ promotes recombination competence, UvrD participates in MMR and NER, UvrD and DinG participate in the resolution of TRCs and restrain excess recombination, and Rep supports replisome movement. The net outcome reflects the physiological balance between transcriptional load, DNA damage burden, and SOS-mediated gene expression.

### Implications of genome organization, TRCs, and SOS induction for antibiotic tolerance

Our results reveal a mechanistic link between *rrn* operon copy number and survival during quinolone exposure. Strains with fewer *rrn* operons survived NAL treatment better, likely because higher basal TRC levels increase SOS induction and precondition cells for recombinational repair. This effect disappeared in *lexA*(Ind^−^) cells, confirming that SOS induction underlies the survival difference. Since quinolones such as NAL kill by trapping gyrase cleavage complexes and converting replication collisions into DSBs (Drlica and Zhao 1997), survival under these conditions depends heavily on RecA-mediated recombination and SOS-driven repair.

Thus, the enhanced survival of the *rrnC*^+^ strain under NAL treatment mirrors antibiotic tolerance, where physiological state—rather than resistance mutations—provides temporary protection (Balaban et al. 2019). Thus, genome organization, TRC frequency, and SOS regulation together shape bacterial responses to DNA-damaging antibiotics, revealing a mechanistic link between endogenous replication stress and transient antibiotic tolerance.

## Concluding remarks

In summary, helicase function during severe TRC at highly transcribed *rrn* operons is not intrinsically protective or deleterious but is contingent upon the recombination landscape shaped by SOS induction (Fig. 6, Supplementary Table 5). SOS induction shifts the balance between productive TRC resolution and harmful recombination interference mediated by UvrD and DinG, largely by controlling cellular RecA abundance and therefore the capacity for homologous recombination repair. RecQ consistently acts as a promoter of SOS induction and productive recombination, whereas Rep functions as a constitutive, SOS-independent accessory helicase supporting replisome progression. These findings also reveal that TRC-driven stress can influence antibiotic resilience; by triggering the SOS response, TRCs at *rrn* loci can enhance a cell's ability to withstand DNA-damaging agents such as quinolones. Ultimately, these results emphasize that bacterial helicase functions must be interpreted within their regulatory context, where the shifting balance between replication dynamics and recombination control determines genome stability and cellular survival.

## Supplementary Material

jkag205_Supplementary_Data

## Data Availability

Strains are available upon request. The authors affirm that all data necessary for confirming the conclusions of the article are present within the article, figures, and tables. Supplemental material available at G3 online.
