# Supplementary material for: The SOS response acts as a regulatory switch that balances helicase anti-recombination with transcription–replication conflict resolution activities in Escherichia coli
Source: G3 (Bethesda). 2026 Jul 29;16(10):jkag205. doi: 10.1093/g3journal/jkag205 (PMC13643760; doi:10.1093/g3journal/jkag205)
Supplement: Supplementary file 1 [file jkag205_supplementary_data.zip › Supplementary_Figure_1_G3-2026-406811.pdf]

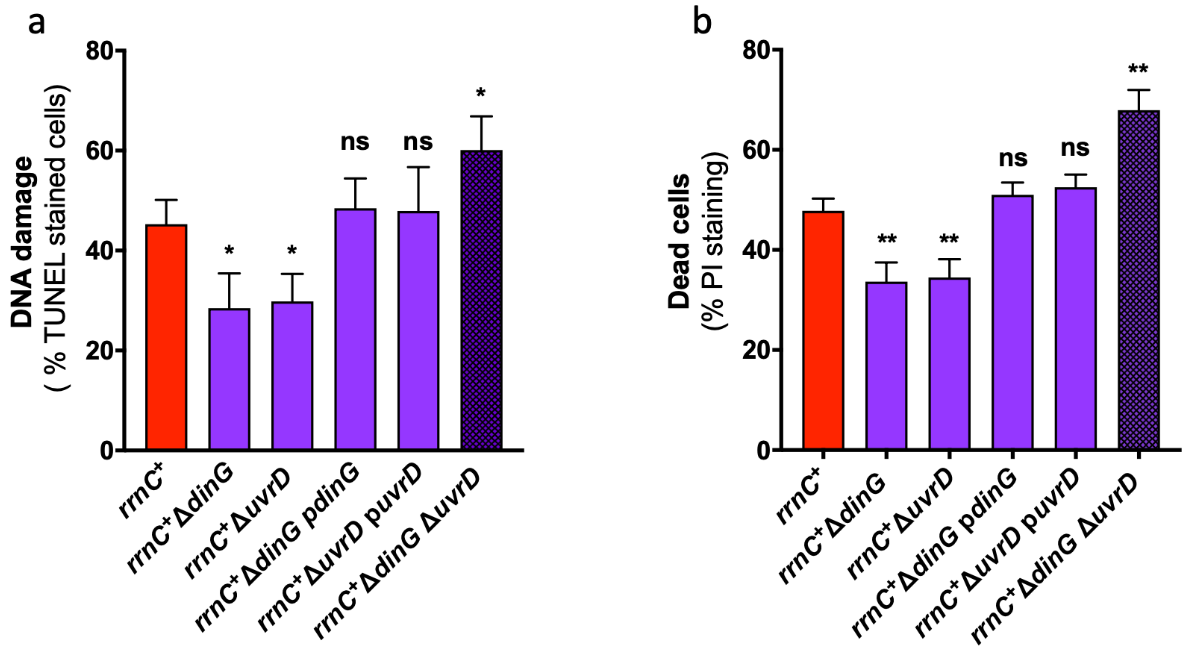

Supplementary Figure 1: DNA breakage and viability of the *rrnC*+ strain and its helicase mutant derivatives. (a) DNA breakage in lag phase cells, quantified using a TUNEL assay. (b) Dead cells were detected using propidium iodide (PI) staining and flow cytometry. Bars represent the mean value ± SD of three independent experiments. Asterisks indicate significant differences according to a t-test: *P < 0.05, **P < 0.01. NS: not significant.
