# Supplementary material for: The SOS response acts as a regulatory switch that balances helicase anti-recombination with transcription–replication conflict resolution activities in Escherichia coli
Source: G3 (Bethesda). 2026 Jul 29;16(10):jkag205. doi: 10.1093/g3journal/jkag205 (PMC13643760; doi:10.1093/g3journal/jkag205)
Supplement: Supplementary file 1 [file jkag205_supplementary_data.zip › Supplementary_Figure_2_G3-2026-406811.pdf]

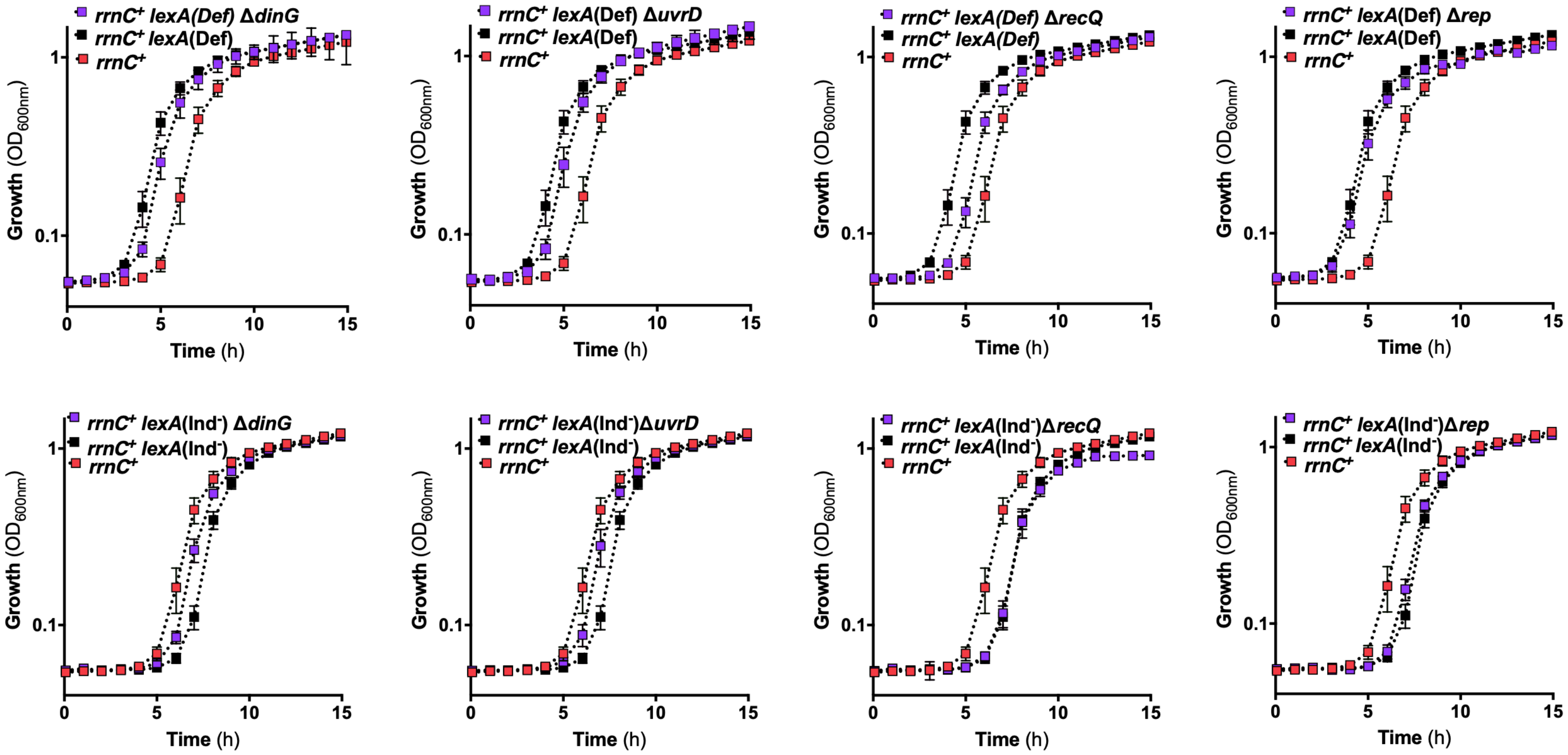

Supplementary Figure 2: Growth curves of *rrnC*⁺, *rrnC*⁺ *lexA*(Def) (upper row) and *rrnC*⁺ *lexA*(Ind⁻) (bottom row) and their helicase mutant derivatives. Each data point represents the mean value (± SD) of three independent experiments. Related to Fig. 2gh and Supplementary Table 2.
