# Supplementary material for: The SOS response acts as a regulatory switch that balances helicase anti-recombination with transcription–replication conflict resolution activities in Escherichia coli
Source: G3 (Bethesda). 2026 Jul 29;16(10):jkag205. doi: 10.1093/g3journal/jkag205 (PMC13643760; doi:10.1093/g3journal/jkag205)
Supplement: Supplementary file 1 [file jkag205_supplementary_data.zip › Supplementary_Figure_3_G3-2026-406811.pdf]

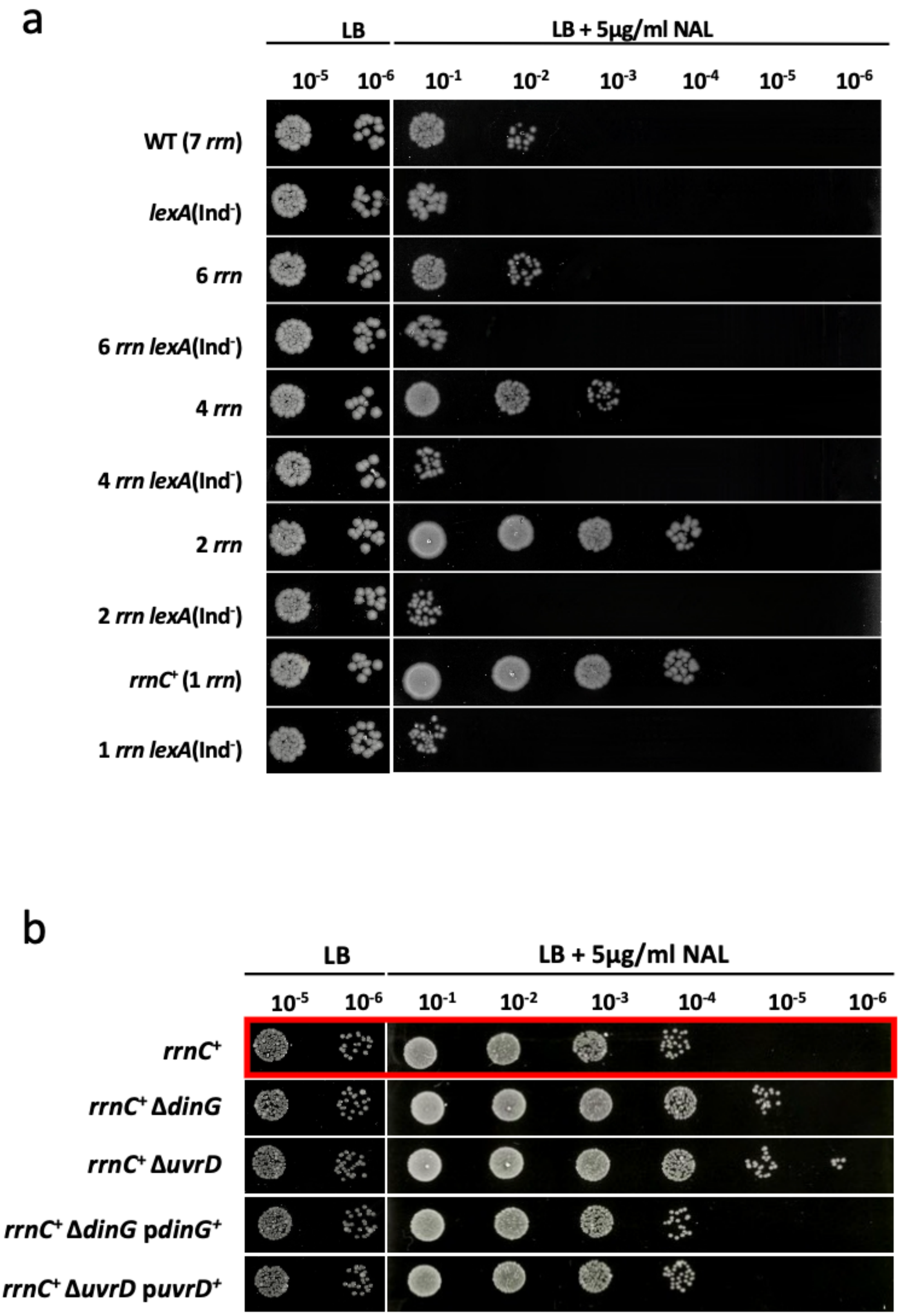

Supplementary Figure 3: (a) Survival of strains having different number of *rrn* operons and their *lexA*(Ind^{-}) derivatives treated with nalidixic acid. (b) Impact of complementation with plasmid-expressed functional *dinG* and *uvrD* on the survival of *rrnC*$^{+}$ Δ*dinG* and *rrnC*$^{+}$ Δ*uvrD* strains treated with nalidixic acid. Overnight liquid cultures were serially diluted and spotted onto LB agar plates containing or not containing 5 µg/ml NAL. The results are representative of at least three independent experiments.
