# Supplementary material for: The SOS response acts as a regulatory switch that balances helicase anti-recombination with transcription–replication conflict resolution activities in Escherichia coli
Source: G3 (Bethesda). 2026 Jul 29;16(10):jkag205. doi: 10.1093/g3journal/jkag205 (PMC13643760; doi:10.1093/g3journal/jkag205)
Supplement: Supplementary file 1 [file jkag205_supplementary_data.zip › Supplementary_Table_1_G3-2026-406811.pdf]

Supplementary Table 1: Bacterial strains

Name	*E. coli* strains	Genotype	Sources
SF1	MG1655 : 7 *rrn* (WT)	*rph*-1λ^{-}	Lab collection
SF2	1*rrn* (*rrnC*$^{+}$)	*rph*-1λ^{-}Δ(*rrsH-aspU*)794::FRT Δ(*rrfG-rrsG*)791::FRT Δ(*rrfF-rrsD*)793::FRT Δ(*rrsA-rrfA*)792::FRT Δ(*rrsB-rrfB*)790::FRT Δ(*rrsE-rrfE*)789::FRT [ptRNA67]= p15a *aadA*	[1]
SF3	2 *rrn*	*rph*-1λ^{-}Δ(*rrsH-aspU*)794::FRT Δ(*rrfG-rrsG*)791::FRT Δ(*rrfF-rrsD*)793::FRT Δ(*rrsA-rrfA*)792::FRTΔ(*rrsE-rrfE*)789::FRT [ptRNA67]= p15a *aadA*	[1]
SF4	4 *rrn*	*rph*-1λ^{-}Δ(*rrfG-rrsG*)791::FRTΔ(*rrsA-rrfA*)792::FRT Δ(*rrsB-rrfB*)790::FRT	[1]
SF5	6 *rrn*	*rph*-1λ^{-}Δ(*rrsE-rrfE*)785(::FRT)	[1]
SF6	Δ*dinG*	as SF1 but Δ*dinG*::Kan	This study [2]
SF7	*rrnC*$^{+}$ Δ*dinG*	as SF2 but Δ*dinG*::Kan	This study [2]
SF8	*rrnC*$^{+}$ Δ*dinG* p*dinG*$^{+}$	as SF7 but p*dinG*$^{+}$	This study [2, 3]
SF9	Δ*uvrD*	as SF1 but Δ*uvrD*::Kan	This study [2]
SF10	*rrnC*$^{+}$Δ*uvrD*	as SF2 but Δ*uvrD*::Kan	This study [2]
SF11	*rrnC*$^{+}$Δ*uvrD* p*uvrD*$^{+}$	as SF10 but p*uvrD*$^{+}$	This study [2, 3]
SF12	*rrnC*$^{+}$ Δ*dinG* Δ*uvrD*	As SF6 but Δ*uvrD*::Cm	This study [2]
SF13	Δ*rep*	as SF1 but Δ*rep*::Kan	This study [2]
SF14	*rrnC*$^{+}$Δ*rep*	as SF2 but Δ*rep*::Kan	This study [2]
SF15	Δ*recQ*	as SF1 but Δ*recQ*::Kan	This study [2]
SF16	*rrnC*$^{+}$Δ*recQ*	as SF2 but Δ*recQ*::Kan	This study [2]
SF17	*lexA* (Ind^{-})	as SF1 but *lexA1 malB*::Tn*9*	This study [2]
SF18	*rrnC*$^{+}$ *lexA* (Ind^{-})	as SF2 but *lexA1 malB*::Tn*9*	This study [2]
SF19	Δ*dinG lexA* (Ind^{-})	as SF1 but *lexA1 malB*::Tn*9*	This study [2]
SF20	*rrnC*$^{+}$Δ*dinG lexA* (Ind^{-})	as SF2 but *lexA1 malB*::Tn*9*	This study [2]
SF21	Δ*uvrD lexA* (Ind^{-})	as SF1 but *lexA1 malB*::Tn*9*	This study [2]
SF22	*rrnC*$^{+}$Δ*uvrD lexA* (Ind^{-})	as SF2 but *lexA1 malB*::Tn*9*	This study [2]
SF23	Δ*rep lexA* (Ind^{-})	as SF1 but *lexA1 malB*::Tn*9*	This study [2]
SF24	*rrnC*$^{+}$Δ*rep lexA*(Ind^{-})	as SF2 but *lexA1 malB*::Tn*9*	This study [2]
SF25	Δ*recQ lexA* (Ind^{-})	as SF1 but *lexA1 malB*::Tn*9*	This study [2]
SF26	*rrnC*$^{+}$Δ*recQ lexA* (Ind^{-})	as SF2 but *lexA1 malB*::Tn*9*	This study [2]
SF27	*lexA* (Def) Δ*sulA*	as SF1 but *lexA*::Kan Δ*sulA*	This study [2]
SF28	*rrnC*$^{+}$*lexA* (Def) Δ*sulA*	as SF2 but *lexA*::Kan Δ*sulA*	This study [2]
SF29	Δ*dinG lexA* (Def) Δ*sulA*	Δ*dinG*::Cm; *lexA*::Kan Δ*sulA*	This study [2]
SF30	*rrnC*$^{+}$ Δ*dinG lexA* (Def) Δ*sulA*	*rrnC*$^{+}$ Δ*dinG*::Cm; *lexA*::Kan Δ*sulA*	This study [2]
SF31	Δ*uvrD lexA* (Def) Δ*sulA*	Δ*uvrD*::Cm; *lexA*::Kan Δ*sulA*	This study [2]
SF32	*rrnC*$^{+}$Δ*uvrD lexA* (Def) Δ*sulA*	*rrnC*$^{+}$ Δ*uvrD*::Cm; *lexA*::Kan Δ*sulA*	This study [2]
SF33	Δ*rep lexA* (Def) Δ*sulA*	Δ*rep*::Cm; *lexA*::Kan Δ*sulA*	This study [2]
SF34	*rrnC*$^{+}$Δ*rep lexA* (Def) Δ*sulA*	*rrnC*$^{+}$ Δ*rep*::Cm; *lexA*::Kan Δ*sulA*	This study [2]
SF35	Δ*recQ lexA* (Def) Δ*sulA*	Δ*recQ*::Cm; *lexA*::Kan Δ*sulA*	This study [2]
SF36	*rrnC*$^{+}$Δ*recQ lexA* (Def) Δ*sulA*	*rrnC*$^{+}$ Δ*recQ*::Cm; *lexA*::Kan Δ*sulA*	This study [2]
SF37	*rrnC*$^{+}$ P$_{recA}$-*yfp*	as SF2 but *intC*::P$_{recA}$-*yfp*-Cm	This study [2,4]
SF38	*rrnC*$^{+}$*lexA* (Def) P$_{recA}$-*yfp*	as SF25 but *intC*::P$_{recA}$-*yfp*-Cm	This study [2]
SF39	*rrnC*$^{+}$*lexA* (Ind^{-}) P$_{recA}$-*yfp*	as SF15 but *intC*::P$_{recA}$-*yfp*-FRT	This study [2]
SF40	*rrnC*$^{+}$Δ*dinG* P$_{recA}$-*yfp*	as SF7 but *intC*::P$_{recA}$-*yfp*-Cm	This study [2]
SF41	*rrnC*$^{+}$Δ*uvrD* P$_{recA}$-*yfp*	as SF9 but *intC*::P$_{recA}$-*yfp*-Cm	This study [2]
SF42	*rrnC*$^{+}$Δ*rep* P$_{recA}$-*yfp*	as SF11 but *intC*::P$_{recA}$-*yfp*-Cm	This study [2]
SF43	*rrnC*$^{+}$Δ*recQ* P$_{recA}$-*yfp*	as SF13 but *intC*::P$_{recA}$-*yfp*-Cm	This study [2]
SF44	*recAo*^{c}	*recAo*^{c} *srl*::Tn*10*	Lab collection [5]
SF45	*recAo*^{c} Δ*dinG*	as SF41 but SF6	This study [2]
SF46	*recAo*^{c} Δ*uvrD*	as SF41 but SF8	This study [2]
SF47	*recAo*^{c} Δ*recQ*	as SF41 but SF10	This study [2]
SF48	*recAo*^{c} Δ*rep*	as SF41 but SF12	This study [2]
SF49	*recAo*^{c} *lexA* (Ind^{-})	*recAo*^{c} *srl*::Tn*10*; *lexA1 malB*::Tn*9*	This study [2]
SF50	*recAo*^{c} *lexA* (Ind^{-}) Δ*dinG*	as SF46 but SF6	This study [2]
SF51	*recAo*^{c} *lexA* (Ind^{-}) Δ*uvrD*	as SF46 but SF8	This study [2]
SF52	*recAo*^{c} *lexA* (Ind^{-}) Δ*recQ*	as SF46 but SF10	This study [2]
SF53	*recAo*^{c} *lexA* (Ind^{-}) Δ*rep*	as SF46 but SF12	This study [2]
SF54	2 *rrn lexA* (Ind^{-})	as SF3 but SF14	This study [2]
SF55	*4 rrn lexA* (Ind^{-})	as SF4 but SF14	This study [2]
SF56	6 *rrn lexA* (Ind^{-})	as SF5 but SF14	This study [2]

[1] Quan et al. 2015 (DOI: 10.1534/g3.115.022301). Strains obtained from the Coli Genetic Stock Center, Yale University, New Haven, CT, USA.

[2] Gene deletions transduced from the Keio collection (Baba et al., 2006, DOI: 10.1038/msb4100050) or lab collection using P1 transduction.

[3] p*dinG*$^{+}$ and p*uvrD*$^{+}$ plasmids (Saka et al. 2005, DOI: 10.1093/dnares/12.1.63).

[4] P$_{recA}$-*yfp* reporter was constructed by Marie-Florence Bredèche.

[5] *recAo*^{c} allele carries an A to G base substitution at position -64 within the *recA* gene promoter region, which confers a constitutive *recA* expression.
