# Supplementary material for: The SOS response acts as a regulatory switch that balances helicase anti-recombination with transcription–replication conflict resolution activities in Escherichia coli
Source: G3 (Bethesda). 2026 Jul 29;16(10):jkag205. doi: 10.1093/g3journal/jkag205 (PMC13643760; doi:10.1093/g3journal/jkag205)
Supplement: Supplementary file 1 [file jkag205_supplementary_data.zip › Supplementary_Table_2_G3-2026-406811.pdf]

Supplementary Table 2: Lag time duration and doubling time of the different WT and *rrnC*$^+$ strain mutant derivatives (related to Figs. 1, 2gh and Supplementary Fig. 2).

**WT background**	**Lag time (min) (mean ± SD)**	**t-test**	**Doubling time (min) (mean ± SD)**	**t-test**
**WT**	193.5 ± 2.6	/	37.6 ± 2.2	/
Δ*dinG*	199.1± 7.2	NS	37.8 ± 1.9	NS
Δ*uvrD*	197.6 ± 4.9	NS	38.2 ± 3.1	NS
Δ*recQ*	201.5 ± 4.9	NS	38.4 ± 3.3	NS
Δ*rep*	209.4 ± 7.2	NS	44.3 ± 2.2	*
rrnC$^+$ **background**	**Lag time (min) (mean ± SD)**	**t-test**	**Doubling time (min) (mean ± SD)**	**t-test**
rrnC$^+$	343.1 ± 13.4	/	59.1 ± 4.6	/
Δ*dinG*	284.9 ± 9.8	**	58.3 ± 2.6	NS
Δ*uvrD*	249.5 ± 8.3	***	61.9 ± 4.3	NS
Δ*recQ*	565.2 ± 22.6	***	81.3 ± 4.4	***
Δ*rep*	372.9 ± 15.7	NS	73.2 ± 2.3	***
lexA(Ind$^-$)	416.6 ± 8.3	**	62.5 ± 3.6	NS
lexA(Def)	227.8 ± 8.6	***	56.1 ± 3.7	NS
rrnC$^+$ ***lexA*****(Ind**$^-$**) background**	**Lag time (min) (mean ± SD)**	**t-test**	**Doubling time (min) (mean ± SD)**	**t-test**
lexA**(Ind**$^-$**)**	416.6 ± 8.3	/	62.5 ± 3.6	/
Δ*dinG*	377.8 ± 8.7	*	61.5 ± 5.3	NS
Δ*uvrD*	376.4 ± 13.4	*	60.2 ± 5.9	NS
Δ*recQ*	412.5 ± 12.5	NS	61.1 ± 5.6	NS
Δ*rep*	398.7 ± 24.5	NS	62.4 ± 6.8	NS
rrnC$^+$ ***lexA*****(Def) background**	**Lag time (min) (mean ± SD)**	**t-test**	**Doubling time (min) (mean ± SD)**	**t-test**
lexA**(Def)**	227.8 ± 8.6	/	56.1 ± 3.7	/
Δ*dinG*	258.4 ± 11.0	*	57.4 ± 5.3	NS
Δ*uvrD*	273.3 ± 13.0	*	60.2 ± 4.1	NS
Δ*recQ*	298.6 ± 18.8	*	58.8 ± 5.7	NS
Δ*rep*	244.9 ± 20.4	NS	61.2 ± 2.7	NS

Significant differences for helicase mutants relative to the WT, *rrnC*$^+$, *rrnC*$^+$ *lexA*(Ind$^-$) and *rrnC*$^+$ *lexA*(Def) backgrounds, according to a t-test: *P < 0.05, **P < 0.01, ***P < 0.001, NS: not significant
