# Supplementary material for: The SOS response acts as a regulatory switch that balances helicase anti-recombination with transcription–replication conflict resolution activities in Escherichia coli
Source: G3 (Bethesda). 2026 Jul 29;16(10):jkag205. doi: 10.1093/g3journal/jkag205 (PMC13643760; doi:10.1093/g3journal/jkag205)
Supplement: Supplementary file 1 [file jkag205_supplementary_data.zip › Supplementary_Table_3_G3-2026-406811.pdf]

Supplementary Table 3: Quantification of the survival of WT and its mutant derivatives after 1 h of treatment with 4 µg ml⁻¹ nalidixic acid.

	Strains	Cellular concentration (CFU/ml) (mean ± SD)	Survival T/NT (mean ± SD)	Survival parental strain/mutant t-test
**NT**	WT	1.04e+08 ± 1.03e+06	/	/
	Δ*dinG*	1.10e+08 ± 1.00e+06	/	/
	Δ*uvrD*	1.07e+08 ± 1.53e+06	/	/
	Δ*recQ*	1.05e+08 ± 1.1e+06	/	/
	Δ*rep*	1.11e+08 ± 5.77+05	/	/
	lexA(Ind^-)	1.10e+08 ± 5.00e+06	/	/
	lexA(Ind^-) Δ*dinG*	1.25e+08 ± 1.23e+06	/	/
	lexA(Ind^-) Δ*uvrD*	1.16e+08 ± 2.03e+06	/	/
	lexA(Ind^-) Δ*recQ*	1.17e+08 ± 1.53e+06	/	/
	lexA(Ind^-) Δ*rep*	1.45e+08 ± 1.67e+06	/	/
	lexA(Def)	1.65e+08 ± 3.77+06	/	/
	lexA(Def) Δ*dinG*	1.38e+08 ± 5.00e+06	/	/
	lexA(Def) Δ*uvrD*	1.04e+08 ± 2.30e+06	/	/
	lexA(Def) Δ*recQ*	1.10e+08 ± 2.40e+06	/	/
	lexA(Def) Δ*rep*	1.17e+08 ± 1.83e+06	/	/
**Post NAL treatment**	WT	1.43e+07 ± 2.61e+06	1.38e-01 ± 2.01e-2	/
	Δ*dinG*	1.13e+07 ± 1.98e+06	1.03e-01 ± 3.23e-2	NS
	Δ*uvrD*	1.83e+07 ± 2.11e+06	1.71e-01 ± 1.63e-2	NS
	Δ*recQ*	2.01e+07 ± 3.21e+06	1.91e-01 ± 2.55e-2	NS
	Δ*rep*	1.31e+07 ± 2.23e+06	1.18e-01 ± 1.47e-2	NS
	lexA(Ind^-)	9.91e+06 ± 2.01e+06	9.01e-02 ± 2.41e-2	/
	lexA(Ind^-) Δ*dinG*	1.10e+07 ± 1.11e+06	8.80e-02 ± 2.95e-2	NS
	lexA(Ind^-) Δ*uvrD*	1.13e+07 ± 1.83e+06	9.74e-02 ± 3.01e-2	NS
	lexA(Ind^-) Δ*recQ*	9.31e+07 ± 3.10e+06	7.96e-02 ± 3.21e-2	NS
	lexA(Ind^-) Δ*rep*	1.23e+07 ± 2.16e+06	8.48e-02 ± 3.11e-2	NS
	lexA(Def)	2.33e+07 ± 3.11e+06	1.41e-01 ± 1.01e-2	/
	lexA(Def) Δ*dinG*	2.11e+07 ± 1.45e+06	1.53e-01 ± 2.51e-2	NS
	lexA(Def) Δ*uvrD*	2.13e+07 ± 3.05e+06	2.05e-01 ± 3.23e-2	NS
	lexA(Def) Δ*recQ*	9.93e+06 ± 3.24e+06	9.03e-02 ± 2.71e-2	*
	lexA(Def) Δ*rep*	2.23e+07 ± 3.10e+06	1.91e-01 ± 2.64e-2	NS

Significant differences for each helicase mutants relative to its parental strains (in gray): WT, *lexA*(Ind^-) and *lexA*(Def), according to a t-test: *P < 0.05, NS: not significant
