# Supplementary material for: The SOS response acts as a regulatory switch that balances helicase anti-recombination with transcription–replication conflict resolution activities in Escherichia coli
Source: G3 (Bethesda). 2026 Jul 29;16(10):jkag205. doi: 10.1093/g3journal/jkag205 (PMC13643760; doi:10.1093/g3journal/jkag205)
Supplement: Supplementary file 1 [file jkag205_supplementary_data.zip › Supplementary_Table_4_G3-2026-406811.pdf]

Supplementary Table 4: Growth restart of WT and its mutant derivatives after nalidixic acid treatment calculated from the growth curves (related to Fig. 4).

**WT background**	**Lag time (min) (mean ± SD)**	**t-test**	**Doubling time (min) (mean ± SD)**	**t-test**
**WT**	330.4 ± 11.7	/	39.1 ± 3.5	/
Δ*dinG*	297.2 ± 9.3	*	38.6 ± 2.3	NS
Δ*uvrD*	317.8 ± 15.9	NS	37.8 ± 1.9	NS
Δ*recQ*	417.6 ± 9.6	***	38.8 ± 4.8	NS
Δ*rep*	350.0 ± 7.3	NS	37.9 ± 6.8	NS
***lexA*(Ind$^-$) background**	**Lag time (min) (mean ± SD)**	**t-test**	**Doubling time (min) (mean ± SD)**	**t-test**
***lexA*(Ind$^-$)**	523.9 ± 10.7	/	37.8 ± 5.5	/
Δ*dinG*	298.8 ± 11.8	****	38.4 ± 3.8	NS
Δ*uvrD*	297.2 ± 9.3	****	40.3 ± 1.8	NS
Δ*recQ*	590.8 ± 7.0	***	39.3 ± 3.8	NS
Δ*rep*	417.6 ± 7.2	***	38.6 ± 4.7	NS
***lexA*(Def) background**	**Lag time (min) (mean ± SD)**	**t-test**	**Doubling time (min) (mean ± SD)**	**t-test**
***lexA*(Def)**	262.6 ± 10.5	/	37.7 ± 3.2	/
Δ*dinG*	265.6 ± 7.3	NS	40.3 ± 4.5	NS
Δ*uvrD*	249.4 ± 6.0	NS	38.4 ± 2.5	NS
Δ*recQ*	326.4 ± 10.5	**	42.5 ± 7.5	NS
Δ*rep*	302.6 ± 3.6	**	42.2 ± 3.2	NS

Significant differences for helicase mutants relative to the WT, *lexA*(Ind$^-$) and *lexA*(Def) backgrounds, according to a t-test: *P < 0.05, **P < 0.01, ***P < 0.001, ****P < 0.0001, NS: not significant
