# Supplementary material for: The SOS response acts as a regulatory switch that balances helicase anti-recombination with transcription–replication conflict resolution activities in Escherichia coli
Source: G3 (Bethesda). 2026 Jul 29;16(10):jkag205. doi: 10.1093/g3journal/jkag205 (PMC13643760; doi:10.1093/g3journal/jkag205)
Supplement: Supplementary file 1 [file jkag205_supplementary_data.zip › Supplementary_Table_5_G3-2026-406811.pdf]

Supplementary Table 5: Functional effects of deletions of genes encoding the UvrD, DinG, RecQ, and Rep helicases

Genetic background of the parental strain	Additional mutations	Lag phase duration	Doubling time	DNA Breakage	Cell mortality	SOS Induction	NAL survival	Post-NAL growth restart duration
WT	Δ*uvrD*	NS relative to WT	NS relative to WT	NS relative to WT	NS relative to WT	-	Increased relative to WT	NS relative to WT
	Δ*dinG*	NS relative to WT	NS relative to WT	NS relative to WT	NS relative to WT	-	Increased relative to WT	Shorter relative to WT
	Δ*recQ*	NS relative to WT	NS relative to WT	NS relative to WT	NS relative to WT	-	Decreased relative to WT	Longer relative to WT
	Δ*rep*	NS relative to WT	Longer relative to WT	NS relative to WT	NS relative to WT	-	As WT	NS relative to WT
	lexA (Ind$^-$)	-	-	-	-	-	Decreased relative to WT	-
	lexA (Def)	-	-	-	-	-	Increased relative to WT	-
	recAo^C	-	-	-	-	-	As WT	-
	recAo^C Δ*uvrD*	-	-	-	-	-	Increased relative to WT	-
	recAo^C Δ*dinG*	-	-	-	-	-	Increased relative to WT	-
	recAo^C Δ*recQ*	-	-	-	-	-	As WT	-
	recAo^C Δ*rep*	-	-	-	-	-	As WT	-
lexA (Ind$^-$)	Δ*uvrD*	-	-	-	-	-	Increased relative to *lexA* (Ind$^-$)	Shorter relative to *lexA* (Ind$^-$)
	Δ*dinG*	-	-	-	-	-	Increased relative to *lexA* (Ind$^-$)	Shorter relative to *lexA* (Ind$^-$)
	Δ*recQ*	-	-	-	-	-	As *lexA* (Ind$^-$)	Longer relative to *lexA* (Ind$^-$)
	Δ*rep*	-	-	-	-	-	Increased relative to *lexA* (Ind$^-$)	Shorter relative to *lexA* (Ind$^-$)
	recAo^C	-	-	-	-	-	Increased relative to *lexA* (Ind$^-$)	-
	recAo^C Δ*uvrD*	-	-	-	-	-	Increased relative to *recAo*^{C} *lexA* (Ind$^-$)	-
	recAo^C Δ*dinG*	-	-	-	-	-	Increased relative to *recAo*^{C} *lexA* (Ind$^-$)	-
	recAo^C Δ*recQ*	-	-	-	-	-	As *lexA* (Ind$^-$) *recAo*^{C}	-
	recAo^C Δ*rep*	-	-	-	-	-	As *lexA* (Ind$^-$) *recAo*^{C}	-
lexA (Def)	Δ*uvrD*	-	-	-	-	-	As *lexA* (Def)	NS relative to *lexA* (Def)
	Δ*dinG*	-	-	-	-	-	As *lexA* (Def)	NS relative to *lexA* (Def)
	Δ*recQ*	-	-	-	-	-	Decreased relative to *lexA* (Def)	Longer relative to *lexA* (Def)
	Δ*rep*	-	-	-	-	-	Decreased relative to *lexA* (Def)	Longer relative to *lexA* (Def)
rrnC$^+$	Δ*uvrD*	Shorter relative to *rrnC*$^+$	NS relative to *rrnC*$^+$	Reduced relative to *rrnC*$^+$	Reduced relative to *rrnC*$^+$	Increased relative to *rrnC*$^+$	Increased relative to *rrnC*$^+$	-
	Δ*dinG*	Shorter relative to *rrnC*$^+$	NS relative to *rrnC*$^+$	Reduced relative to *rrnC*$^+$	Reduced relative to *rrnC*$^+$	Increased relative to *rrnC*$^+$	Increased relative to *rrnC*$^+$	-
	Δ*recQ*	Longer relative to *rrnC*$^+$	Longer relative to *rrnC*$^+$	Increased relative to *rrnC*$^+$	Increased relative to *rrnC*$^+$	Reduced relative to *rrnC*$^+$	Decreased relative to *rrnC*$^+$	-
	Δ*rep*	NS relative to *rrnC*$^+$	Longer relative to *rrnC*$^+$	NS relative to *rrnC*$^+$	NS relative to *rrnC*$^+$	Slightly reduced relative to *rrnC*$^+$	As *rrnC*$^+$	-
	lexA (Ind$^-$)	Longer relative to *rrnC*$^+$	NS relative to *rrnC*$^+$	-	-	No induction	Decreased relative to *rrnC*$^+$	-
	lexA (Def)	Shorter relative to *rrnC*$^+$	NS relative to *rrnC*$^+$	-	-	Derepression	Increased relative to *rrnC*$^+$	-
rrnC$^+$ *lexA* (Ind$^-$)	Δ*uvrD*	Shorter relative to *rrnC*$^+$ *lexA* (Ind$^-$)	NS relative to *rrnC*$^+$ *lexA* (Ind$^-$)	-	-	-	Increased relative to *rrnC*$^+$ *lexA* (Ind$^-$)	-
	Δ*dinG*	Shorter relative to *rrnC*$^+$ *lexA* (Ind$^-$)	NS relative to *rrnC*$^+$ *lexA* (Ind$^-$)	-	-	-	Increased relative to *rrnC*$^+$ *lexA* (Ind$^-$)	-
	Δ*recQ*	NS relative to *rrnC*$^+$ *lexA* (Ind$^-$)	NS relative to *rrnC*$^+$ *lexA* (Ind$^-$)	-	-	-	Decreased relative to *rrnC*$^+$ *lexA* (Ind$^-$)	-
	Δ*rep*	NS relative to *rrnC*$^+$ *lexA* (Ind$^-$)	NS relative to *rrnC*$^+$ *lexA* (Ind$^-$)	-	-	-	Increased relative to *rrnC*$^+$ *lexA* (Ind$^-$)	-
rrnC$^+$ *lexA* (Def)	Δ*uvrD*	Longer relative to *rrnC*$^+$ *lexA* (Def)	NS relative to *rrnC*$^+$ *lexA* (Def)	-	-	-	Decreased relative to *rrnC*$^+$ *lexA* (Def)	-
	Δ*dinG*	Longer relative to *rrnC*$^+$ *lexA* (Def)	NS relative to *rrnC*$^+$ *lexA* (Def)	-	-	-	As *rrnC*$^+$ *lexA* (Def)	-
	Δ*recQ*	Longer relative to *rrnC*$^+$ *lexA* (Def)	NS relative to *rrnC*$^+$ *lexA* (Def)	-	-	-	Decreased relative to *rrnC*$^+$ *lexA* (Def)	-
	Δ*rep*	NS relative to *rrnC*$^+$ *lexA* (Def)	NS relative to *rrnC*$^+$ *lexA* (Def)	-	-	-	Decreased relative to *rrnC*$^+$ *lexA* (Def)	-

- : not measured

NS : not significant
